# Nucleic Acid and Immunological Diagnostics for SARS-CoV-2: Processes, Platforms and Pitfalls

**DOI:** 10.3390/diagnostics10110866

**Published:** 2020-10-23

**Authors:** Avinash Premraj, Abi George Aleyas, Binita Nautiyal, Thaha J Rasool

**Affiliations:** Camel Biotechnology Center, Presidential Camels and Camel Racing Affairs Centre, Department of the President’s Affairs, P.O. Box 17292, Al Ain 17292, UAE; avinash.p@precamels.ae (A.P.); abi.a@precamels.ae (A.G.A.); binita.n@precamels.ae (B.N.)

**Keywords:** COVID-19 testing, molecular diagnostics, immunological testing, RT-qPCR, ELISA, pool PCR, lateral flow assay, rapid assay

## Abstract

Accurate diagnosis at an early stage of infection is essential for the successful management of any contagious disease. The coronavirus disease 2019 (COVID-19), caused by the Severe Acute Respiratory Syndrome Coronavirus 2 (SARS-CoV-2) virus is a pandemic that has affected 214 countries affecting more than 37.4 million people causing 1.07 million deaths as of the second week of October 2020. The primary diagnosis of the infection is done either by the molecular technique of RT-qPCR by detecting portions of the RNA of the viral genome or through immunodiagnostic tests by detecting the viral proteins or the antibodies produced by the host. As the demand for the test increased rapidly many naive manufacturers entered the market with novel kits and more and more laboratories also entered the diagnostic arena making the test result more error-prone. There are serious debates globally and regionally on the sensitivity and specificity of these tests and about the overall accuracy and reliability of the tests for decision making on control strategies. The significance of the test is also complexed by the presence of asymptomatic carriers, re-occurrence of infection in cured patients as well as by the varied incubation periods of the infection and shifting of the viral location in the host tissues. In this paper, we review the techniques available for SARS-CoV-2 diagnosis and probable factors that can reduce the sensitivity and specificity of the different test methods currently in vogue. We also provide a checklist of factors to be considered to avoid fallacious practices to reduce false positive and false negative results by the clinical laboratories.

## 1. Introduction

The coronavirus disease 2019 (COVID-19), caused by the Severe Acute Respiratory Syndrome CoronaVirus 2 (SARS-CoV-2 virus) is believed to have originated in Dec 2019 at Wuhan city, China [1,2] and spread to 214 countries of the world affecting more than 37.4 million people and causing more than 1.07 million deaths as of the second week of October 2020. The virus named as SARS-CoV-2 belongs to the order *Nidovirales* and family *Coronoviridae* and subfamily *coronavirinae* that includes four genera. The SARS-CoV-2 belongs to the genus Betacoronavirus and subgenus Sarbecoviruses and is related to other human coronaviruses like SARS-CoV and Middle East Respiratory Syndrome Coronavirus (MERS-CoV) and carries an enveloped, non-segmented positive strand RNA genome of 30 kb. The viral genome codes for four structural proteins and sixteen nonstructural proteins [3]. The virus enters the host cells through the angiotensin converting enzyme-2 (ACE2) receptor using the receptor-binding domain of the S protein and causes a battery of symptoms. The symptoms are manifested 2 to 14 days after exposure and include fever, cough, shortness of breath, chills, muscle pain, pressure in the chest, headache, sore throat, confusion, bluish lips on the face, anosmia and impaired taste [4].

To enable management of a pandemic outbreak, rapid and accurate diagnosis is of paramount importance. Initial clinical/symptomatic investigations depended on blood count, coagulation profile, serum biochemical tests like Liver Function Test (LFT) and Renal Function Test (RFT), creatine kinase, lactate dehydrogenase, and other electrolytes. Respiratory specimens including nasal and pharyngeal swabs, bronchoalveolar lavage fluid, sputum, or bronchial aspirates were tested for common viruses, including influenza, avian influenza, respiratory syncytial virus, adenovirus, parainfluenza virus, SARS-CoV and MERS-CoV using Reverse Transcription quantitative PCR (RT-qPCR) assays approved by the China Food and Drug Administration. Routine bacterial and fungal examinations were also performed.

The World Health Organization (WHO) declared COVID-19 as a pandemic on 11 March 2020 [5]. Even though the mortality rate is around 3% in different populations so far infected, the speed at which the infection is spreading is alarming. The only way to check out its spread in the absence of an effective vaccine or a therapeutic agent is by breaking the human to human contact chain, which is being practiced through quarantine-lockdown by most of the countries affected. Only a small fraction of the infected individuals develop serious symptoms, such as severe pneumonia, Acute Respiratory Distress Syndrome (ARDS), and sepsis, leading to the death of the infected patient.

The etiological agent of COVID-19 is diagnosed in the clinical laboratory either through the presence of selected genes of the viral genome, selected viral proteins or by the antibodies produced by the infected host. The major genes targeted for the detection of the viral genome are the nucleocapsid protein gene (*N*), the spike protein gene (*S*), the envelope protein gene (*E*), RNA dependent RNA polymerase (*RdRp*) and part of the major *ORF1a/b*. The majority of the countries that are practicing population quarantine are developing strategies to come to normalcy for which appropriate sensitive and specific diagnostic tests are essential.

Serological tests depend on the detection of viral antigenic proteins or the antibodies produced by the infected hosts. The viral antigen detection is similar to the molecular techniques, directly detecting the presence of viral particles. However, the test is complexed by the sampling procedure and the presence of viral particles in the samples collected. On the other hand, antibody detection is much simpler as the serum or plasma from the venous blood of the patient is used for the test and it does not vary with the sampling procedure as source tissue is liquid. An added advantage is that there is no sample processing step like RNA isolation or cDNA synthesis. The test can reliably confirm people subjected to infection with or without symptoms even though cross-reactivity with closely related coronaviruses may give rise to a false-positive result. Further, the presence of neutralizing antibodies in infected people and more or less the same level of antibodies among people with mild or severe clinical symptoms [6] can indicate the level of herd immunity in a population. The serological test also helps in identifying silent infection retrospectively.

This review is a compilation of all existing diagnostic methods for detection of SARS-CoV-2 along with their comparative merits and demerits so as to provide a ready reference to clinical laboratories engaged in COVID-19 diagnosis. We have reviewed the various nucleic acid and immunological tests and platforms available for SARS-CoV-2 diagnosis, probable factors that can reduce the sensitivity and specificity of the tests and the critical care to be taken for avoiding fallacious results.

## 2. Nucleic Acid Amplification Techniques for SARS-CoV-2 Diagnosis

A number of molecular techniques have been developed for the detection of viral genome signatures. The most important technique or the gold standard being recommended by most of the countries is Reverse Transcription quantitative PCR (RT-qPCR) targeting different gene/genes. Majority of the tests use an *E gene* targeted RT-qPCR as a preparative test followed by *RdRp* and/or Nucleocapsid *N gene* as confirmatory tests [7].

Reverse Transcriptase quantitative PCR (RT-qPCR) for SARS-CoV-2 diagnosis: Reverse Transcriptase quantitative PCR (RT-qPCR) is the first choice and globally accepted diagnostic assay for the screening of the SARS-CoV-2 virus. The pandemic nature of the SARS-CoV-2 outbreak of 2020 has sparked RT-qPCR testing of the virus on an unprecedented scale globally. Kary Mullis, the originator of the Polymerase Chain Reaction (PCR) might not have thought even in his wildest dreams that the technique he invented will become the most widely used diagnostic test by mankind. The unforeseen and urgent demand for SARS-CoV-2 testing has stretched diagnostic RT-qPCR resources and personnel to the limit and warranted quick deployment of new testing facilities and personnel to the scene. The major RT-qPCR assays available for SARS-CoV-2 detection are listed in Table 1. We focus on diverse PCR based diagnostic methods available and critical technical factors affecting the SARS-CoV-2 diagnosis and provide suggestions for improving the accuracy and sensitivity of the tests.

### 2.1. Specimen Types and Sampling Procedure

#### 2.1.1. Specimen Types for Testing

The success of the diagnostic procedure depends on the collection of appropriate specimens from the right anatomic location/organ at the right time of infection. The initial studies on the bio-distribution of SARS-CoV-2 in various patient samples during the outbreak in the Hubei, Shangdong and Beijing in China revealed the highest PCR positive rates in bronchoalveolar lavage (BAL) (93%), followed by sputum (72%), nasal swabs (63%), pharyngeal swabs (32%) and feces (29%) [8]. Another study revealed that viral samples in the sputum and throat swabs peaked around 5–6 days after onset of the symptoms [9].

#### 2.1.2. Respiratory Sampling

Respiratory sampling is the most common and widely accepted method for SARS-CoV-2 detection by RT-qPCR and can be broadly divided into upper respiratory and lower respiratory sampling [10]. Upper respiratory sampling includes a nasal swab (NS) taken from the anterior nasal cavity, a nasopharyngeal swab (NPS) taken deep from the posterior mucopharnygeal wall, an oropharyngeal swab (OPS) from around the posterior pharyngeal wall, a lingual swab (LS) taken from the anterior part of the tongue and gargle lavage. Lower respiratory samples include sputum, tracheal aspirates (TA) and bronchoalveolar lavage (BAL) [10].

NS, NPS and OPS are taken with flocked swabs. The upper respiratory sampling is comparatively easier than the lower respiratory sampling but need trained personnel. The discomfort created for the patients by the procedure could induce sneezing or coughing, which can lead to aerosol exposure of the virus [11]. It has been suggested that upper respiratory sample swabs like Nasal (NS) or inner cheek (CS) may be equally sensitive in acute COVID-19 patients and can be effectively taken by patients themselves [11]. It is also suggested that patient should be advised to keep the eyes wide open during NPS collection as it helps to irrigate nasopharyngeal walls with tears that help to increase the viral load in swabs. The general impulsive reaction of the patient is to close the eyes at the time of NPS collection (personal communication).

BAL is reported to have a higher diagnostic value than upper respiratory samples in patients with pneumonia [10]. However, the bio-safety risk of aerosol dispersal of the infective virus cannot be ignored while collecting sputum/BAL samples. Moreover, BAL sample collection requires bronchoscopy and staff with specialized training and is not practical for large scale screening purposes. Tracheal aspirates (TA) samples are collected by intubation and suction from mechanically ventilated patients or tracheotomized patients and the procedure is performed only when absolutely necessary [10]. Hence, upper respiratory samples (nasopharyngeal swabs), which are relatively easier to collect under limited resource settings with minimally trained staff, are the most preferred samples for screening [12]. However, the variation in the viral load of the nasopharyngeal samples should not be ignored [13].

#### 2.1.3. Self-Collected Samples

The widely adapted NPS or OPS swabs testing for SARS-CoV-2 needs interaction of health care workers with patients and requires specialized swabs and personal protective equipment, which are in short supply. This has led to exploration of other types of samples that patients can collect on their own, without discomfort and minimizing the risk of nosocomial infections. Of late, deep throat saliva (DTS) was also reported as an alternative for patient self-collected specimens with less health hazard to workers and without compromising diagnostic results [14]. However, patient training and briefing on the DTS collection procedure is critical as the quality of the DTS specimen depends upon the technique, volume and time of the day of DTS collection. Specimen quality for DTS is hence important considering that viral RNA concentration in DTS is lower than NP and throat swabs [15].

Oral fluid and saliva have also recently been proposed as potential samples that the patients could collect by themselves and had shown similar diagnostic sensitivity to NPS [16,17]. For oral fluid specimens patients are instructed to cough deep three to five times, collect phlegm or secretions in their mouth, and the swab is rubbed on all parts of the oral cavity (inner cheeks, above and below the tongue, gums, teeth and the hard palate) for around 20 s. After this the swab is inserted into a collection tube with a storage media and sent to the testing facility. Coughing is a critical step while collecting the oral fluid specimen for SARS-CoV-2 testing and improperly collected specimens may miss viral detection [16]. Like the DTS, oral fluid specimen collection by patients themselves also warrants proper training through video based instruction or demonstration.

Although self-collected patient samples have advantages like wider reach especially for the elder patients, for risk mitigation of exposure to the virus, the technical quality of specimen collection has to be considered especially for nasopharyngeal and oropharyngeal swabs. In a study in Iran of 50 adult patients, it was revealed that there was discordance in the result between professional-collected and patient-collected samples. Many oropharyngeal and nasopharyngeal swabs that were negative in patient-collected samples were positive when collected by laboratory professionals [18]. In the iCollect Cohort Pilot Study in the USA, 153 participants took part in a specimen self-collection study after receiving kits and instructions for collecting oropharyngeal swab (OPS), saliva, and dried blood spot specimens (DBS). Simple, graphical and easy to understand instructions for self-collection of specimens were provided. In this study more than 96% of the saliva and OPS samples had sufficient quality for laboratory testing. One hundred percent of the OPS samples and 98% of the saliva samples had RNAse P threshold cycle (Ct) values greater than 30, indicating sufficient nucleic acid in the sample for SARS-CoV-2 PCR testing [19].

#### 2.1.4. Variation in the Viral Load

Virological assessment of individual SARS-CoV-2 positive samples by two independent laboratories in Germany provided insights into the viral load in patients [6]. Swabs were taken from nine patients starting from day one of symptoms and all swabs were positive from days one to five, with average viral load being 6.76 × 10^5^ copies per whole swab with maximum load detected on the fourth day being 7.11 × 10^8^. After day five the load of samples decreased with an average viral load of 3.44 × 10^5^ copies per swab and a detection rate of 39.93%. The last sample that was tested positive was taken 28 days after onset of symptoms. The study also revealed that there was no significant difference in viral load or detection rates between nasopharyngeal and oropharyngeal swab samples. When compared to the SARS virus, peak viral loads were detected much earlier for SARS-CoV-2 (before day 5 for SARS-CoV-2 and days 7–10 for SARS). The concentration of virus was also much higher for SARS-CoV-2 (1000 times more than SARS). In the German study, sputum samples had an average viral load of 7 × 10^6^ copies/mL and a maximum of 2.35 × 10^9^ copies/mL and the decline in viral load was slow compared to swabs. In two-thirds of the patients (6/9), sputum and stool remained positive for viral RNA by PCR for more than three weeks [6].

In a study of 213 patients in China, sputum samples provided a higher rate of accuracy in positive diagnosis (75 to 90%), when compared to nasal swabs (53 to 73%) during the first 14 days after the onset of symptoms [20]. However, based on current reports, nasopharyngeal swabs are adequate specimens considering they have the highest viral loads among different sample types. The CDC recommends only using nasopharyngeal swabs, although oropharyngeal swabs are also acceptable specimens [21].

#### 2.1.5. Sample Collection, Storage and Transport

Proper sampling techniques using appropriate swabs are essential for high-quality upper respiratory specimens, otherwise it will lead to false RT-qPCR negatives. CDC recommends only synthetic fiber swabs with plastic or wire shafts and strongly advises against using calcium alginate swabs or swabs with wooden shafts as they can inactivate some viruses and inhibit PCR reactions (CDC, 2020). The swab should be inserted through the nostrils parallel to the palate until resistance is encountered. Swabs should reach a depth equal to distance from the nostrils to outer opening of the ear. After collection, the swabs must be placed in the viral transport media (VTM) or Universal Transport Media (UTM), and stored and transported as per the guidelines recommended by CDC/WHO. For swabs in VTM, storage at 2–8 °C is recommended for up to 72 h and −70 °C for longer duration. Considering the fact that many of the mass samplings for SARS-CoV-2 is done at outdoor/mobile/makeshift sampling stations outside proper clinical facilities, care should be taken to store and transport the swabs at the recommended temperatures to the testing facilities where RNA isolation is carried out to prevent false negatives due to sample degradation. Appropriate biosafety protocols for the COVID-19 specimen samples from collection, transport and processing have been suggested to prevent laboratory-associated SARS-CoV-2 infection [22].

### 2.2. RNA Isolation

Isolation of RNA is the initial step of any quantitative reverse transcriptase PCR assay. This is one of the most critical steps determining the reproducibility and biological relevance of the results. RNA, unlike DNA, is highly susceptible to degradation. So the sample storage, handling and RNA isolation steps have to be carried out with optimized protocols minimizing the loss of RNA due to degradation at each step. The isolated RNA must be of high quality, without any nucleases or substances that may inhibit cDNA synthesis. Among clinical samples, mammalian blood, in particular, the heme compound is a well-known PCR inhibitor. RNA isolation from respiratory samples like sputum is reported to have yielded false-negative results due to challenges in isolating RNA from mucus containing sputum [23]. Additional preprocessing/homogenization of mucus containing sputum has been reported to improve the sensitivity for respiratory viral pathogens by reverse transcriptase qPCR, especially when using automated RNA isolation platforms. Proteinase K and dithiothreitol (DTT) treatment prior to RNA isolation significantly improved RNA quality and Threshold cycle (Ct) value in RT-qPCR for the detection of influenza A virus in sputum [23]. Dithiothreitol (DTT) reduces the disulfide bonds in mucins and effectively homogenizes mucus. For the RT-qPCR detection of Middle East Respiratory Syndrome coronavirus (MERS-CoV), another *Betacoronavirus*, the proteinase K and DNase I method of homogenizing sputum reduced false negatives in spiked sputum samples [24]. Hence, similar homogenization methods need to be employed for SARS-CoV-2 in sputum samples to reduce false negatives.

The pandemic nature of the SARS-CoV-2 outbreak has resulted in an unprecedented demand for viral RNA based diagnostics and has resulted in a shortage of RNA isolation kits/reagents. In such a scenario, various research groups have come up with direct RT-qPCR assays skipping the RNA isolation process. Some of these tests have accurately identified 92% clinically known positive samples without the need for RNA isolation [25,26]. In critical situations where lack of RNA isolation kit severely hampers the testing process, such methods could be adopted with necessary caution so that the testing process does not come to a standstill.

#### RNA Isolation Quality Control

Positive and negative controls in RNA isolation are of paramount importance due to the huge numbers of samples tested, wide variety of specimen types, and different types of RNA isolation systems. To ensure sufficient human RNA is obtained from the specimen, RNAse *P* mRNA is used in RT-qPCR as an internal house keeping control. Positive RNA controls can be spiked samples with synthetic SARS-CoV-2 RNA or previously validated positive samples. The demand for RNA isolation from a huge number of samples resulted in the adaptation of a wide variety of RNA isolation systems—automated and manual and some of which may lead to aerosol contamination of samples due to improper use. A previously validated negative patient sample serves as negative extraction control for RNA isolation to rule out such issues [27]. A no-template control without any RNA (viral transport medium/phosphate buffered saline/water) is also recommended during RNA extraction, and this control should not give any amplification (RNase P or SARS-CoV-2 targets).

### 2.3. cDNA Synthesis

#### 2.3.1. Reverse Transcription Systems

For the detection of RNA viruses like SARS-CoV-2 reverse transcription qPCR is recommended as the most sensitive method. The isolated total RNA (viral RNA and the host RNA) is reverse transcribed into a cDNA first using reverse transcriptase enzyme. PCR or qPCR is performed using the cDNA as a template to exponentially amplify the target gene of interest. In the beginning, this was a two-step process with the cDNA synthesis performed separately first using the isolated RNA, and aliquots of the cDNA subsequently used for PCR or qPCR. Reverse transcriptase of viral origin from Avian Myeloblastosis Virus (AMV-RT) and Moloney Murine Leukemia Virus (MMLV-RT) were the two enzymes widely used. AMV-RT has a higher reaction temperature, thereby reducing problems associated with RNA secondary structure. MMLV-RT has lower RNAse H activity than AMV-RT and hence has better yield and longer size of cDNA products [28]. Newer generation engineered reverse transcriptase available in the market feature higher thermostability, enhanced fidelity, diminished RNAse H activity and enhanced processivity. Currently, many vendors offer one-step reverse transcriptase PCR solutions in a blend of reverse transcriptase enzymes and Taq DNA polymerases in an optimized reaction buffer. RNA and gene-specific primers are added into the reaction mix, and there is no need to change buffers or add additional reagents between the reverse transcription and PCR amplification step [28]. Such one-step single tube RT-qPCR assays are convenient for diagnostic assays, reducing chances of cross-contamination and PCR product contamination.

#### 2.3.2. Primers for Reverse Transcription

Reverse transcription can be carried out by using different primers—oligo-dT, random primers or gene-specific. The choice of the primer depends on the type of RNA, cDNA yield, and specificity. Use of oligo-dT primer for RT will make the cDNA synthesis for mRNA and not for ribosomal RNA or viral transcripts or RNA transcripts with significant secondary structure. Random primers bind at multiple portions of the RNA transcript and are not specific. However, they yield the most amount of cDNA and can bind with a wide variety of RNA like rRNA, viral RNA and RNA with secondary structure [29]. The use of random primers will ensure that both the SARS-CoV-2 viral RNA and human RNA (for the host control RNA like RNAseP) are reverse transcribed and the same cDNA can be used for qPCR.

#### 2.3.3. RNase P as Control

The use of *RNase P* gene control serves as an important sample extraction quality control. Along with the viral RNA, the patient’s RNAse P mRNA is also isolated during the RNA isolation step. The RNAse P mRNA is reverse transcribed into cDNA and the RNAse P Primers amplify a fragment of this gene, which is then detected by real-time PCR. As per the CDC guidelines, all clinical samples tested should have Ct values less than 40 for RNase P in RT-qPCR. Failure to detect *RNase P* gene within the prescribed limits may indicate improper RNA extraction, RNA degradation/loss, absence of sufficient human cellular material due to improper collection, reagent or equipment malfunction. It may be noted that the RNase P gene control PCRs are run in parallel with the SARS-CoV-2 and not multiplexed in the same PCR reaction along with the SARS-CoV-2 target genes as per CDC/WHO recommendations. The relative high abundance of human RNase P RNA in the sample when compared to SARS-CoV-2 viral RNA may cause reduced sensitivity of SARS-CoV-2 target genes when multiplexed.

#### 2.3.4. Positive and Negative Controls

In this unprecedented testing on a global scale for SARS-CoV-2, it is critical to maintain proper quality control standards in RT-qPCR. The assays are mostly performed in multi-well plates and are prone to aerosol contamination. RT-PCR negative control is usually nuclease-free water and a proper negative control should not give any amplification signals. In-vitro synthesized RNA transcripts based on the SARS-CoV-2 are used as positive control samples and are expected to give Ct (threshold cycle) in the recommended ranges by different assay kits [27]. Any amplification in the negative control sample for PCR should be a red flag and the entire set of sample results should be suspect.

### 2.4. RT-qPCR for SARS-CoV-2

#### 2.4.1. Primers for SARS-CoV-2 Detection

For RT-qPCR, CDC recommends primer-probe sets for two different regions of the SARS-CoV-2 *nucleocapsid* gene (*N1* and *N2*). A third primer-probe set for the human *RNase P* gene serves as a control for RNA extraction and cDNA synthesis. The WHO recommends the use of a different set of SARS-CoV-2 genes; RNA-dependent RNA polymerase *(RdRP)* and envelope (*E*) genes [30]. These real-time PCR primers/probe sets recommended by CDC and WHO are highly sensitive and specific for SARS-CoV-2 and have minimal cross-reactivity with other strains of coronaviruses [31]. CDC and WHO have provided a list of commercial SARS-CoV-2 kits that have been validated extensively and for Emergency Use Authorization (EUA) by various regulatory agencies. However, the primers and probe sets need to be experimentally validated using appropriate SARS-CoV-2 positive control sets for each lot. If the primer and probe are custom synthesized, these also need to be validated for sensitivity, specificity and stability.

#### 2.4.2. Comparative Sensitivity of the Primer Probe Sets Used for SARS-CoV-2

A recent comparative study from Yale School of Medicine with different primer/probes for SARS-CoV-2 detection revealed that sensitive primer-probe sets were E-Sarbeco, HKU-ORF1, and 2019-nCoV_N1. The RdRp-SARS had the lowest sensitivity that could be attributed to a mismatch in the reverse primer [32]. In another independent study with seven different primer-probe sets and a single real-time PCR assay system also, the *E* gene primer-probe set [30] and the N2 primer set of CDC was found to be most sensitive than others [33]. The insufficient sensitivity of the RdRp, when compared to *N* gene, is also reported by SARS-CoV-2 testing centers in Korea, who suggest that the *N* gene is 7–43 fold more sensitive [34].

#### 2.4.3. Amplifications Conditions

Reaction conditions for COVID-19 RT-qPCR depend on the target gene, primers, reverse transcriptases, *Taq* DNA polymerases, and thermal cyclers used. The Charite paper suggests the following PCR cycling conditions: reverse transcription at 55 °C for 10 min and initial denaturation at 95 °C for 3 min, followed by 45 cycles of 95 °C for 15 s, 58 °C for 30 s [7]. CDC recommends a slightly different protocol with initial denaturation at 95 °C for two minutes, followed by 45 cycles of 95 °C for 3 s, 55 °C for 30 s [35]. It must be noted that the extension-annealing step in the 45 cycles of qPCR, where the data capture occurs, is set at different temperatures as per Charite and CDC. CDC suggests a lower temperature of 55 °C for this step when compared to 58 °C of Charite. Commercial kits available for detection of SARS-CoV-2 use different combinations of primers in a multiplex or singleplex, and use different types of Reverse transcriptases/*Taq* polymerases with different reaction conditions. These kits are validated at specific conditions recommended by the manufacturers based on the assay design/reagents used. Hence, users should follow instructions and reaction conditions recommended for the individual kits/reagents they are using and should not follow a general protocol for PCR amplification.

#### 2.4.4. Data Analysis

Even before starting the RT-qPCR run, the data capture settings in the qPCR software should be accurately set to capture the data in real-time. CDC provides clear guidelines on instrument setup, entering fluorescent detector, sample setup and reaction conditions in the real time PCR software [35]. The target genes, samples, control, etc., should be clearly defined and assigned to the respective wells. Care should be taken to correctly define the fluorescent probe reporter and quencher used by the assay for the different targets. Different commercial kits use different target reporter and quencher combinations, in singleplex/multiplex and these have to be entered correctly for each assay.

The Ct (threshold cycle) value is the most commonly used parameter for reporting diagnostic qPCR results. Ct is the cycle when the sample fluorescence exceeds the background fluorescence by a chosen threshold [29]. Most real-time PCR softwares has inbuilt algorithms for determining baseline fluorescence using the background signal from all the wells and estimating the automatic threshold. The threshold for each target should be manually checked for each run and carefully selected so that it should intersect the fluorescent amplification curve at the exponential phase of the amplification well above any background noise [35].

CDC provides clear guidelines on the interpretation of assays and results with threshold and Ct values. The No Template Control (NTC) without the RNA for all the primer and probe combinations should not have fluorescence amplification curves that cross the threshold line. If any NTC reactions show fluorescence that crosses the threshold level, contamination needs to be suspected and assays repeated. The SARS-CoV-2 positive control samples should have a Ct value of less than 40, well above any background fluorescence. Reagent failure or primer or probe integrity needs to be suspected in case of any Ct value beyond 40 or lack of well-defined amplification curves for the positive controls. The human RNAse P (RP) controls are also expected to show amplification curves that cross the threshold within the 40 cycles. Failure to detect RNAse P could be due to poor collection method yielding insufficient cellular material, improper nucleic acid extraction from the specimen and instrument/reagent/assay malfunction. Ideally in an assay, SARS-CoV-2 positive control samples and RNAse P control should have Ct values <40 and NTC controls should not have fluorescence amplification curves that cross the threshold line within 40 cycles. A clinical specimen is considered clear positive when all the SARS-CoV-2 target genes and RNAse P have Ct value < 40. Additional scenarios with Ct values and interpreting them are discussed in detail in the CDC guidelines [35].

### 2.5. Other Nucleic Acid Detection Technologies

Apart from the RT-qPCR for SARS-CoV-2, which is considered the gold standard and most widely used, there are many other nucleic acid detection technologies for the virus that are summarized below.

### 2.6. Reverse Transcriptional Loop-Mediated Isothermal Amplification (RT-LAMP) for SARS-CoV-2 Detection

Loop-mediated Isothermal Amplification (LAMP) is a cost-efficient one-tube isothermal nucleic acid amplification technique carried out at a single temperature that amplifies DNA fragments of interest with high specificity and sensitivity. In the original method, four different primers, recognizing six distinct regions of the DNA were used along with a DNA polymerase with high strand displacement activity [36]. The LAMP reaction can be monitored visually using colorimetric or fluorescent dyes. LAMP assay being isothermal does not require expensive thermal-cyclers or real-time PCR machines. Furthermore, the LAMP assay is robust, simpler and easy to set up and has a similar sensitivity as conventional PCR or qPCR. This makes LAMP an attractive diagnostic procedure for the low-cost field deployment of various pathogens in developing countries.

Although RT-qPCR is currently the gold-standard in SAR-CoV-2 diagnosis, the protocols are time-consuming, laborious and need specialized expensive equipment and trained personnel. Well validated RT-LAMP assays for SARS-CoV-2 would be simpler, cost-efficient, speedy and equally sensitive alternatives to RT-qPCR. For the detection of RNA viruses like MERS Coronavirus, reverse transcription has been successfully coupled with LAMP to create a one-pot RT-LAMP assay that had been validated [37] much before the outbreak of SARS-CoV-2.

Using the sequences from 103 SARS-CoV-2 genomes, a LAMP assay was developed by a group in China using primers specific to *ORF1ab* and *S* genes. The assay did not have cross-reactivity to 60 other respiratory pathogens and was validated with 130 COVID-19 positive clinical RNA samples already tested by qPCR. The assay showed 100% sensitivity and specificity and gave results within 30 min [38]. Researchers in South Korea have reported another RT-LAMP assay targeting the nucleocapsid *(N)* protein gene with a detection limit comparable to RT-qPCR. The assay was evaluated on 156 clinical samples with sensitivity of 100% and specificity of 98.7% [39]. Another RT-LAMP assay called iLACO (isothermal LAMP-based method for COVID-19) was also developed using a new nucleic acid dye called Genefinder with enhanced fluorescent signal and sensitivity to detect up to 10 copies of ORF1ab gene. The iLACO test was validated on 248 COVID-19 positive RNA samples and detected 223/248 (89.9%) correctly. The 25 iLACO false-negative samples when checked by Taqman RT-qPCR were found to contain very low amounts of viral RNA with Ct values above 35 [40].

Recently LAMP has been coupled with nanopore sequencing to develop LAMPore Assay for detection of the SARS-CoV-2 virus. The LAMPore SARS-CoV-2 is a multiplex assay that targets three different regions of the virus genome (*ORF1a*, *N*, and *E* genes) and a human actin control. Multiple samples (up to 96) are amplified by multiplexed barcoded loop-mediated amplification and SARS-CoV-2 detected by real-time nanopore sequencing in less than two hours on a single MinION flow cell [41].

### 2.7. CRISPR (Clustered Regularly Interspaced Short Palindromic Repeats)-Cas (CRISPR-Associated) Based Molecular Diagnostics

CRISPR (clustered regularly interspaced short palindromic repeats)-Cas (CRISPR-associated) systems in prokaryotes have evolved as an adaptive immune system against invading nucleic acids and this technology has been widely adapted for genome engineering in most living organisms [42]. Different CRISPR based pathogen detection platforms have also been developed utilizing the specific cleavage preferences of different Cas enzymes. The scope of CRISPR associated techniques in COVID-19 detection was also explored by different laboratories so as to find out a faster and reliable test for COVID-19.

#### 2.7.1. SHERLOCK

SHERLOCK (specific high-sensitivity enzymatic reporter unlocking) technology combines isothermal pre amplification with Cas13 to detect single molecules of RNA or DNA at attomolar levels and has been adapted to detect infectious viruses like Dengue and Zika. A field-deployable multiplex viral diagnostic platform was developed by integrating SHERLOCK with HUDSON (Heating Unextracted Diagnostic Samples to Obliterate Nucleases) protocol for viral diagnosis from bodily fluids with minimum equipment and sample processing [43]. A STOP (SHERLOCK Testing in One Pot) assay for detecting SARS-CoV-2 suitable for point-of-care use was developed by Sherlock Biosciences and Broad Institute of MIT. This test detects the *N* gene of SARS-CoV-2 in three simple steps at a sensitivity of 100 viral genomes per reaction and does not need separate viral extraction with specialized equipment. The virus is lysed quickly to release the RNA from the patient sample, followed by RNA detection using STOP Covid and finally visualization of the result using a lateral flow dipstick in 70 min [44]. This kit was given Emergency Use Authorization (EUA) by the FDA for the detection of the virus on 6 May 2020.

#### 2.7.2. DETECTR

Another parallel developed method, DNA Endonuclease-Targeted CRISPR Trans Reporter (DETECTR), uses Cas12a ssDNase activation with isothermal amplification for rapid and specific detection of human viral pathogens with attomolar sensitivity [45]. A SARS-CoV-2 DETECTR based reverse transcription and isothermal amplification using loop-mediated amplification (RT–LAMP) targeting the *N* and *E* genes was developed and validated by Mammoth Biosciences and the University of California [46]. This rapid (<40 min) and visual assay on lateral flow platform eliminates the need for bulky equipment and can detect up to 10 copies of viral RNA per μL of RNA isolated from nasopharyngeal swabs.

#### 2.7.3. FELUDA

This method uses Cas 9 systems from the bacterium *Francisella novicida* (FnCas9) that has been reported to have higher target specificity and negligible affinity with off-targets, when compared to widely used *Streptococcus pyogenes* Cas9 systems [47]. Utilizing the highly specific FnCas9 mediated DNA interrogation and subsequent cleavage, a single nucleotide variant (SNV) detection system called FnCas9 Editor Linked Uniform Detection Assay (FELUDA) was developed by researchers in India. Recombinase Polymerase Amplification (RPA) was coupled with FELUDA to develop a field-deployable lateral flow assay targeting the *NSP8* regions of SARS-CoV-2 for rapid diagnosis [48].

#### 2.7.4. CARMEN-Cas13 System

A microfluidic system for massive multiplexed simultaneous detection of pathogens was developed by the Broad Institute of MIT and Harvard utilizing the specificity of the SHERLOCK Cas13. The CARMEN (Combinatorial Arrayed Reactions for Multiplexed Evaluation of Nucleic acids) platform uses microwell array chips having 177,840 microwells [49]. In this system, viral RNA extracted from the samples is amplified by PCR or Recombinase Polymerase Amplification (RPA). After amplification, each sample is given a unique color code by adding a fluorescent dye. The detection mixture, which is also unique color-coded, consists of Cas13, a guide RNA targeting a specific viral pathogen, and a cleavage reporter. Uniquely color-coded detection sets for 169 pathogenic viruses are multiplexed. The samples and detection sets are emulsified, pooled, and loaded on a chip with many microwells. Each microwell can accommodate two droplets. Nanoliter droplets of CRISPR nucleic acid detection reagents self-organize in microwell array to pair with droplets of amplified samples under an electric field. If a particular detection droplet meets its specific viral target sequence in a sample droplet with the same microwell, a fluorescent signal is produced and is detected by the fluorescent microscope. The color codes of droplets are used for identifying the contents in each microwell. Each massive capacity Chip (mChip) can have >4500 cRNA target pairs in statistical replicates to detect pathogens at attomolar sensitivity. The CARMEN was not developed originally for SARS-CoV-2 or other coronaviruses. While the CARMEN paper was in review, SARS-CoV-2 occurred, and the researchers quickly developed a SARS-CoV-2 assay and incorporated it into the pan-viral assay panel.

### 2.8. Truenat–Affordable Chip-Based Portable PCR

TrueNat is a recent test developed by Indian scientists by adapting the test already in vogue for pulmonary tuberculosis to COVID-19. TrueNat is a chip-based portable PCR test developed for quick and affordable molecular pathogen detection for use in low infrastructure health facilities in developing countries. BigTec Laboratories, Bangalore, India, developed this technology and earlier adapted this in 2014 for early Tuberculosis detection [50]. The system consists of a battery-operated portable micro PCR called TrueNatMTB and Trueprep MAG system for DNA extraction from samples. TrueNAT SARS-CoV-2 has 100% sensitivity and specificity and has no cross-reactivity with other respiratory pathogens. The limit of detection for the TrueNAT SARS-CoV-2 is 407 genome copies/mL. Although the TrueNat technology lacks the throughput of the conventional PCR, its affordability, portability, ease of use and test interpretation makes it a very attractive field deployable solution for COVID-19 screening in the developing world.

### 2.9. Pooled Testing for Population Screening by RT-qPCR

The World Health Organization recommends aggressive testing as one of the main strategies against the COVID-19 pandemic. The overwhelming demand for mass screening by RT-PCR testing for the virus made the global healthcare system struggle with a shortage of testing kits and trained personnel [51]. To expand the testing to a larger population with limited resources, researchers have been considering pooling the samples to scale-up the magnitude of testing. Pool testing involves combining several samples from individuals into a single tube (pool) and the pool sample tested. Only if the pool sample tests positive, the samples are tested separately to identify individuals. Pool testing not only significantly increases the testing capacity but also saves testing resources and time.

One of the first pool PCR testing for SARS-CoV-2 was done in Israel in March 2020. Researchers pooled individually isolated RNA samples that were earlier confirmed as SARS-CoV-2 positive or negative and tested the pools by RT-qPCR. They found that a single clinical SARS-CoV-2 RNA sample can be consistently detected in a pool of up to 32 samples with an estimated false-negative rate of 10% [52]. In the same study, it was noted that as the number of negatives samples in the pool increases, the pooled sample reaches the PCR threshold (Ct) later as expected from diluted samples. Another pool PCR study in Germany using pooled RNA, revealed that up to 30 RNA samples can be pooled to scale up capacity without compromising diagnostic accuracy. This study also pointed to a lowering on the Ct values in pooled samples and suggested the possibility that border line single samples might evade detection in large pools [53]. They suggested that when a pool is large and positive (30 samples) then it is advisable to divide it into three smaller sub-pools (10 samples). The positive sub-pool is then tested individually. Using this strategy, they could test 1191 samples with only 267 tests to detect 23 positive individuals [53]. Sample pooling strategies was implemented at the RNA extraction stage in a study of 184 samples in Israel. No loss of assay sensitivity and accuracy was observed when samples were pooled up to eight samples each, and tested in parallel individually [54].

*In-silico* analysis, using an application called Shiny, estimated the optimum pool size to be five specimens to detect at an assay sensitivity of 95% or 100% and 100% specificity, when the virus prevalence rate is set at 5%. Twenty-five experimental pools created with one known positive specimen and four negative specimens were positive with pooled Ct values with an average difference of 2.45 of the individual specimen Ct values and range of difference from 0 to 5.03. Using this five specimen pool strategy they tested 60 samples using only 22 extractions/PCR tests and identified two positive individuals. They estimated that when the rate of infection of SARS-CoV-2 is less than 10% in the population, the five specimen pool strategy can increase the testing efficiency by 133% and reduce the number of tests by 57% [55].

Mass testing by real-time qPCR for SARS-CoV-2 is laborious, expensive, and has led to a global shortage of diagnostic reagents. With the number of infections on the rise, and a large number of asymptomatic people also to be tested, pool testing will provide a strategy to extend the testing with the existing resources. In countries like India, with a huge number of people, pool testing is adopted by some states and the Indian Council of Medical Research (ICMR) has provided an advisory on using pooled samples (Indian Council of Medical Research, Department of Health Research, 2020). As per the ICMR guidelines, pool PCR testing is recommended for use in areas of low positivity (<2%) and pooling size has to be kept to a maximum of five samples to avoid over dilution leading to false negatives.

Although pool PCR is an interesting strategy to increase the testing scope and improve efficiency with limited resources, it has to be carried out with utmost care by competent testing facilities, without compromising the sensitivity and accuracy.

### 2.10. Environmental Sampling for SARS-CoV-2

The primary transmission of the SARS-CoV-2 virus is believed to be through direct contact and respiratory droplets. However, aerosol transmission and indirect-contact based transmission is also presumed to be involved in the rapid spread of the disease. In a study in Israel, viral RNA could be detected in 52.7% surface samples from surroundings of symptomatic COVID-19 patients in isolation units and 38% from surfaces in quarantine hotels of asymptomatic and mild COVID-19 patients [56]. Potential surfaces with high positivity of RNA included floor, faucet handle, bed side table, bed rails and door handles in hospitals. In hotel public spaces, elevator button panels also had a high RNA positivity in surface swabs. Surface samples were obtained with sterile cotton tipped applicator swabs, whereas air samplers with gelatin membrane filters were used to collect air samples [56]. In a recent study from a hospital in China, toilet surfaces were aerosol dominated with the detection of SARS-CoV-2 in the hospital environmental samples [57]. The SARS-CoV-2 is detected in feces with a median duration of 22 days (even longer than respiratory samples) and the virus is presumed to survive several days out of the body in waste-water. During the March–April COVID-19 outbreak in Paris, the increase in the level of SARS-CoV-2 detected in waste-water was followed by the number of fatal cases reported in the region. This points towards the importance of sewage–waste-water monitoring as a non-invasive warning approach to monitor the level of SARS-CoV-2 and alert communities [58].

## 3. Immunological/Serological Tests for SARS-CoV-2

In the midst of the COVID-19 outbreak, clamor for serological tests also increased either for antibody or antigen detection [59]. Serological or immunological tests come in different formats; the most common type being the ELISA, but the one with utmost demand at present is the Lateral Flow Assay (LFA) test because of its rapidness and ease of testing.

### 3.1. Enzyme Immunoassays

ELISA kits for SARS-CoV-2 antibody detection made by different vendors have been certified by various regulatory agencies. However, for the detection of SARS-CoV-2 antigen only limited ELISA kits are available in the market. ELISA based serological tests can be employed to detect viral antigen in the clinical samples, such as swab, lung lavage, and sputum, or it can be employed to detect the presence of antibodies in the plasma or serum. These tests currently available differ in many respects such as the antigen used, detection method used and the solid matrix employed for capturing antigen/antibody, etc. Apart from well-established mature technologies, novel methods, such as microfluidic chips, plasmonic fiber-optic absorbance biosensor, and field-effect transistor (FET)-based biosensing devices, are being investigated by different laboratories [60,61,62,63]. A representative sample list of currently available ELISA test Kits for COVID-19 is given in Table 2.

### 3.2. Classical Indirect ELISA

Several firms have produced ELISA tests to detect SARS-CoV-2 antibodies based on the classical indirect ELISA method. In indirect ELISA the antigen is coated on ELISA plates to capture virus-specific antibodies, which in turn are detected by secondary conjugated antibodies. Most of the kits available use recombinant spike protein or nucleocapsid protein as the capture antigen. Based on the antigen used to capture, the sensitivity and specificity of the ELISA can vary widely [13]. Some ELISA kits use plates coated with both the spike and nucleocapsid proteins to detect antibodies against both the proteins and increase sensitivity. ELISA detecting IgG (91.9%) was found to have better specificity than IgA (73%) antibodies [64]. Therefore, ELISA to detect IgG is more specific for diagnostics and has high potential for monitoring vaccinated or exposed population. ELISA for IgG gave consistent results if sampling was done 14 or more days after the onset of symptoms [65].

### 3.3. Solid-Phase Antibody Capture ELISA

These are mostly used for the detection of specific subtypes of antibodies such as IgM, IgG or IgA in the serum. Polystyrene microwell strips are pre-coated with antibodies directed against the specific subtype (IgM, IgG, IgA) of human immunoglobulin proteins. During the first incubation after addition of specimen (patient’s serum/plasma), specific subtype antibodies will be captured inside the wells. After washing out all the other substances of the specimen and other antibody subtypes, the antibody subtype-specific to SARS-CoV-2 captured on the solid phase is detected by the addition of SARS-CoV-2 antigen conjugated to the enzyme or other reporter assays [66].

### 3.4. Double Antigen ELISA

Third generation ELISA is known for its higher sensitivity and specificity [67]. Here, patient serum or plasma is added to polystyrene micro wells pre coated with recombinant SARS-COVID-2 antigen and during the first incubation, the specific SARS-COVID-2 antibodies will be captured inside the wells if present. The micro wells are then washed to remove unbound serum proteins and nonspecific antibodies. Second recombinant SARS-COVID-2 conjugated to the enzyme or other reporter is added. During the second incubation the conjugated antigen will bind to the captured antibody inside the wells. The micro wells are then washed to remove unbound conjugates and readout is taken with an appropriate system [68].

### 3.5. ELISA Employing Novel Solid Surfaces—(Bead-Based Automated ELISA)

The classical ELISA employs plastic micro well plates with polystyrene surface to immobilize the antigen. Majority of ELISA kits available in the market are of this kind. This ELISA format was there in the diagnostic field for a long time therefore the procedure has become standardized and most labs are well equipped to carry out the task in terms of equipment and training. However, these are not easily amenable to automated high throughput systems that process hundreds of samples a day, which is the urgent need in the face of the pandemic. Novel formats use magnetic beads or polystyrene beads as a solid matrix to immobilize antigen or antibody depending on the assay. Apart from high throughput, this method allows multiplexing the assays thereby reducing sample volume required. Shorter turnaround time is the main advantage of these automated systems [69].

### 3.6. Different Detection Methods

#### 3.6.1. ELISA Using Chemiluminescence Detection Methods

ELISAs in the market differ in the methods used for detection. The detection methods of ELISA have undergone huge improvements from the traditional enzyme-mediated colorimetric method. Novel automated systems use a more sensitive chemiluminescence detection method, which offers 100 fold more sensitivity and allows automation. Chemiluminescence systems have a detection limit of up to 170pg/mL of the analyte. Abbott chemiluminescence immunoassay is the only system approved by the EU for the detection of COVID-19 antibodies [69]. MAGLUMI CLIA analyzers and DZ-Lite analyzers use chemiluminescence based methods to detect virus specific IgG and IgM in the patient serum [70]. Different variants of the system that use plastic microwell or magnetic microparticles are also available. Roche uses a variant known as electrochemiluminescence, which is claimed to be more sensitive and has a shorter turnaround time [69].

#### 3.6.2. Fluorescence Based ELISA

Fluorescent labels instead of enzymes allow for multiplexed ELISAs, which enable estimation of antibodies against more than one antigen in a single assay or more than one antigen in the matrix. Conjugation with fluorophores with different spectral properties enables quantification of different analytes in a single reaction thus enabling gain on the information in limited turnaround time. The fluorescent immunoassay uses SARS-CoV-2 Ag, covalently linked to polystyrene microspheres. The microspheres are then mixed with serum to bind SARS-CoV-2 antibodies present. Non-bound antibodies are washed away and then bound antibodies are allowed to react with a biotinylated secondary anti-human immunoglobulin reagent (specific for IgM, IgA, and IgG). After washing, microspheres are mixed with phycoerythrin labeled streptavidin and bound antibody is detected with the Luminex cytometer, or other instruments. The fluorescence measured is proportional to the amount of anti-SARS-CoV-2 antibodies in the serum [71].

#### 3.6.3. AlphaLISA

AlphaLISA^®^ immunoassays are designed for the detection and quantification of target molecules in biological samples. These chemiluminescence no-wash assays are ideally suited for miniaturization and automation. It relies on amplified luminescent proximity homogeneous assay. In a sandwich AlphaLISA assay the target is captured by a biotinylated antibody bound to streptavidin-coated donor beads and a second antibody conjugated to AlphaLISA acceptor beads. The binding of the two antibodies to the analyte brings donor and acceptor beads into proximity. Laser irradiation of donor beads at 680 nm generates singlet oxygen, triggering a cascade of chemical events in nearby acceptor beads, which results in a chemiluminescence emission at 615 nm. In competitive AlphaLISA immunoassays, a biotinylated analyte bound to streptavidin donor beads is used with an antibody conjugated to AlphaLISA acceptor beads. AlphaLISA has higher sensitivity and shorter turnaround time as it removes washing steps [72]. However, no vendor has started marketing such an assay for SARS-CoV-2 diagnostics until today.

### 3.7. Antigens Used In Antibody Detection ELISA

The SARS-CoV-2 virus codes for around 20 proteins including 16 nonstructural and 4 structural proteins. The body will potentially produce antibodies against all these proteins and the serum will contain antibodies against all these viral proteins. However, these differ in their abundance and suitability to differentiate from SARS-CoV-2 from other coronaviruses infecting humans. Most of the kits use spike protein (S), or nucleoprotein (N) as antigen. The nature of antigen used has an important bearing on the sensitivity and specificity of the ELISA. The antigen chosen should be able to detect and differentiate from closely related viruses, in this case, common coronaviruses (229E, NL63, OC43) or SARS-CoV (HKU-1) [73]. It has been shown that the nucleocapsid protein-based ELISAs are inferior to spike protein-based ELISAs in discriminating infection with SARS-CoV from other human coronaviruses, [13,73,74] but has been found to have higher sensitivity. The RBD region of spike protein has been shown to differentiate SARS-CoV-1 and SARS-CoV-2 antibodies [73,75]. Immunoassay using the RBD region as an antigen has higher specificity [13]. While selecting the ELISA test kit and interpreting the results, the nature of antigen used should be considered as the most important factor.

## 4. Lateral Flow Tests (LFT)

The lateral flow tests or immunochromatographic tests sometimes called lab on a chip, contains all reagents required to carry out the test in a simple plastic cassette. The principle behind LFA is that a liquid sample containing the analyte of interest moves by capillary action through various zones of the polymeric membrane, on which the molecules that can bind with the analyte are attached or impregnated at a specific location. One of the major advantages of this technique is that no elaborate sample processing step is needed, which is the main drawback of other tests such as ELISA or RT-qPCR. Other advantages of lateral flow testing kits are low cost, simple single-step procedure, requirement of small sample volume, ability to be deployed at the point of care, uniformity of sample used, etc. An added advantage of LFT is the long shelf life of the cassette without refrigeration [76].

### 4.1. Antigen Detection LFA/Immunochromatographic Tests

Immunochromatographic tests for antigen are simple tests, where a nitrocellulose test strip is impregnated with a reporter (colloidal gold or fluorescent nanoparticle or magnetic particles) conjugated to the antibody. In the rapid CoV-2 test, anti-SARS-CoV-2 specific monoclonal antibody either against viral spike protein or nucleocapsid protein is used to impregnate the nitrocellulose filter. The test strip is then dipped in the clinical sample (swab, laryngeal lavage or sputum) and mixed with a buffer. As the liquid moves through the membrane by capillary action, the viral antigen present in the sample reacts with the antibody and forms a complex. The complex is then captured by the second antigen-specific antibody immobilized at the detection line. LFA works on a similar principle of an immunochromatographic test but the sample suspended in the buffer is added by dropper to the sample pad.

### 4.2. Precautionary Measures to Avoid Erroneous Results with Antigen Detection LFA Tests

#### 4.2.1. Sampling

The detail of sampling for antigen testing is similar to the one used for RT-qPCR and has been mentioned under the Molecular Testing. Sampling is a critical part of any diagnostic assay. For SARS-CoV-2, the WHO recommends a nasopharyngeal swab as the ideal sample. However, throat swabs, sputum, and tracheal aspirates are also being used as clinical samples. The sample collection should be done by appropriately trained personnel. Moreover, the abundance of the virus may vary depending on the stage of the infection. RT-qPCR studies have shown that the virus is abundant in the nasopharyngeal swabs during the initial stages of infection but can be detected in anal swabs also at later stages. However, the patient may become ELISA negative for NP samples at the later stages of infection. Therefore, the anatomical site of sample collection for antigen testing is determined by the stage of the infection.

#### 4.2.2. Sample Storage and Transportation

LFAs are supposed to be carried out at the point of care sites. Therefore, storage may not be a critical factor. If samples are transported it should be done under the guidelines of WHO. There is little information available on the effect of different storage conditions on the outcome of Ag LFAs for SARS-CoV-2 detection.

#### 4.2.3. Sample Application

The amount of sample being tested is a critical feature determining the sensitivity of any test. Sampling and sample application are considered as the Achilles heel of LFAs. In the case of the LFAs, the sample volume rarely exceeds 30 μL. Most often the LFAs are supplied as a kit with the dropper or other dispensing mechanism. The volume of the sample is the primary factor determining the flow rate along the membrane, which in turn determines the sensitivity and specificity of the test [77]. Lack of appropriate training can lead to improper sample dispensing and reduction in sensitivity.

#### 4.2.4. Factors Influencing Antigen Detection LFAs

The antibody used to detect antigen is the most critical part of the assay. The antibody used should have high differentiating ability and high affinity to the antigen. Generally monoclonal antibodies are used to increase specificity and avoid false positive reactions. The antibody chosen should be able to detect all variants of the virus. Manufacturers have not given any details regarding the antibodies used in the test (Table 2).

### 4.3. Antibody Detection LFAs

**Figure 1:**
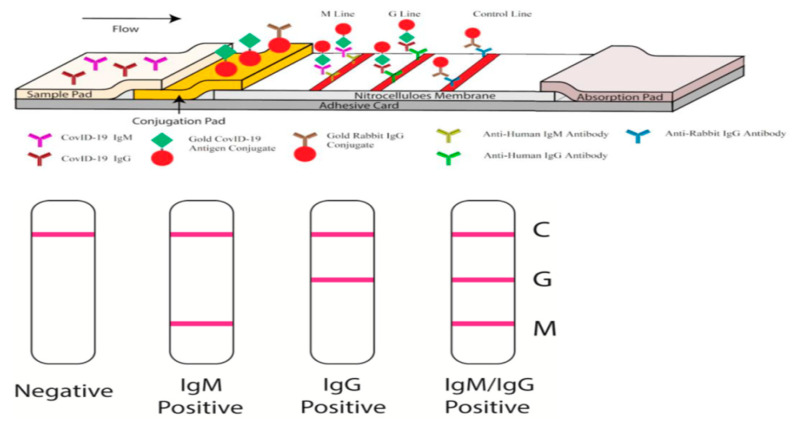
Diagrammatic representation of antibody detection by Lateral Flow Assays (LFAs)—(Image credit https://www.dtnews.it).

These tests are used to detect SARS-CoV-2 specific antibodies in the serum, blood or other biological fluids. The viral antigen conjugated with appropriate reporters such as colloidal gold, fluorescent/magnetic nanoparticle is impregnated in the membrane. Antibody present in the sample interacts with the conjugated antigen while moving through the membrane. LFA for detection of SARS-CoV-2 currently available in the market follows the broad pattern as shown in Figure 1.

The number of test kits in the market is huge and the regulatory mechanisms in many countries are not adequate to safe guard from substandard kits (Table 2—Section 2). Early investigations reported very low sensitivity and specificity levels to many of these kits in many countries [65,78,79].

The antigen used is the most critical part of antibody detection assays. The nature of the antigen used in the commercial kits has not been revealed by many manufacturers. Sensitivity also varies with the antigens used for capturing the antibodies. Most often LFAs use partial proteins or peptides instead of whole antigens. When using such partial antigens many epitopes are lost, especially the conformational epitopes [76]. Therefore, the preservation of the native secondary structure is important while selecting the antigen for the test kit. This can be compromised during production, purification process and storage of antigen and the assay reagents used. Detergents and stabilizing agents are usually incorporated to preserve proteins and increase solubility, which can lead to loss of protein secondary structure and epitopes. Many LFA developers also use short peptides as antigen for better stability and uniformity at the expense of losing some epitopes. Thus peptide antigen leads to non-detection of some of the conformational epitopes thereby removing a subset of antibodies out of the detection spectrum. Further, the conjugation of antigen with reporter dye can induce stearic hindrance leading to modification of epitopes.

### 4.4. Precautionary Measures to Avoid Erroneous Results with Antibody Detection LFA Test

One of the major advantages of antibody detection LFA is avoidance of sampling error as liquid tissues such as blood, serum or plasma are used, which ensures the uniformity of the sample. Recent SARS-CoV-2 LFA kits use finger prick blood as the sample, which removes storage or transportation induced degradation of the samples. However, the person administering the test should be trained properly to avoid common mistakes like using clotted blood and suboptimal sample volume.

Different antibody subtypes appear at different times post infection in an infected patient. IgM antibodies appear around 5–9 days post infection followed by IgG by days 10–14 [80]. Therefore, the test should be carried out based on the stage of infection/the day of post symptoms and the sensitivity of the tests [80]. However, tests that have the potential to differentiate between SARS-CoV-1 and SARS-CoV-2 are to be preferred in countries where CoV-1 was prevalent earlier.

There can be a variation in sensitivity of the LFA kits based on the batch, transportation and storage. The representative samples should be evaluated with known positive and negative sera. Such quality evaluation should be done when:

- A new operator uses the kit;
- A new lot of test kits is used;
- A new shipment of kits is used;
- The temperature used during storage of the kit falls outside of 2–30 °C;
- The temperature of the test area falls outside of 15–30 °C;
- To verify a higher than expected frequency of positive or negative results;
- To investigate the cause of repeated invalid results;
- A new test environment is used (e.g., natural light vs. artificial light).

### 4.5. Detection or Reporter Methods Used in LFAs

The most common LFA comes with a visual read out, eliminating the need of any equipment. Nevertheless, few drawbacks such as very limited sensitivity, individual variation in reading, inability to quantify and difficulty in electronic record keeping are associated with LFA. In order to overcome these drawbacks, assays are being made available using dedicated strip readers. The kits with strip reader facility employ colorimetric, fluorescent or magnetic reporters. The availability of fluorescent nanoparticles such as europium has hastened the adoption of this technique [77]. These methods are claimed to offer better sensitivity, repeatability, removal of individual variations and allow easy electronic record keeping. After application of samples the test strip of the chip is placed in the reader, which is most often dedicated and calibrated for the specific chemistry (colorimetric, florescent or magnetic) and make. The method is considered to bring down the cost of LFAs further as it avoids the use of expensive gold colloid. Although theoretically these reader based assays should have higher sensitivity and specificity, independent comparison in clinical settings is yet to be reported.

### 4.6. Comparison Between ELISA and LFA

Data from the controls are essential to verify the accuracy and reliability of any diagnostic test. This is to ensure integrity and stability of reagents and robustness of the test process. ELISA and RT-qPCR have positive and negative controls incorporated, whereas no such controls are available in LFAs. The control line provides data regarding the optimum flow through the sample pad. Moreover, there is no control incorporated into each assay, which assures the stability of reagents. The lack of proper control is a major drawback of the LFA. [81,82,83]. Some LFAs do provide positive and negative control sera (e.g., Cellex Inc.) to be used as quality control, but these are not run on the same cassette and hence cannot substitute for true positive and negative controls. Most of the LFA kits available in the market do not provide any quality control reagents, information of the antigen used or even information regarding sampling and result interpretation.

### 4.7. Take Away From Serological Test Results

The results of serological tests either positive or negative should be interpreted with care, taking into account the epidemiological factors in the play. Primarily, the record of onset of symptoms during which the samples were collected need to be available to the person deciding on the result. A false negative Ag or Ab tests can occur during the early stages of infection even if sampling errors are omitted. A false negative test can be provided by a test with low sensitivity, which is a case reported by many LFA test kits. False positive antibody reactions are a real possibility where previous SARS-CoV-1 outbreaks had been reported especially with kits using cross-reacting antigens like E gene protein.

An antibody test provides limited information about the infection status of the individual. The earliest antibody reported is day five after onset of symptoms; however, the virus can be detected in nasal swabs even five days prior to the appearance of symptoms [84]. Therefore, absence of antibody cannot be considered as negative status for a person. On the contrary, positive antibody statuses do not indicate an active infection either, as antibody titer remains in the body long after the virus has been cleared. Moreover, the presence of antibody does not indicate the person is not shedding the virus or is immune to further infection. Virus shedding has been reported in cases even after becoming antibody positive. Therefore, a RT-PCR test is necessary to determine if the person is negative or positive for COVID-19. Moreover, the robustness of the antibody response depends on the immune fitness of the individual as there are reports of late or weak antibody responses [68,85,86].

The LFAs or ELISA tests do not provide data regarding protective immunity of an individual. In general, LFAs or ELISA do not provide any information regarding the presence of virus neutralizing antibodies. In order to obtain such data, a virus neutralization test needs to be done, which is a laborious and time consuming test requiring specialized laboratories [73]. Moreover, data so far do not conclusively prove protection from reinfection in survived individuals. Serological tests allow screening of a large population in a very short period of time at minimal cost. Even with all the drawbacks, serological tests are the most cost effective method for epidemiological surveillance of a population where decisions regarding specific individuals are not taken into account.

### 4.8. Potential Pitfalls in COVID-19 Testing

Inadequate/improper sampling: Improper sampling technique may result in low amounts of biological specimen collected for RNA isolation, which may lead to false-negative results. CDC recommends synthetic fiber swabs with plastic or metal shafts [21]. Swabs containing calcium alginate or wooden shafts are known to contain PCR inhibitory substances that can lead to false negative PCR results. After collection, it must be ensured to disperse the collected material completely in the viral transport medium in the container.

Improper handling, storage and transport of samples: Swabs must be stored in containers with recommended Viral Transport Medium (VTM) free of PCR inhibitors. Storage or transport at improper temperatures will lead to degradation of the sample and lead to false-negative results [91].

Improper processing of sputum: Sputum is collected as a specimen in some cases for testing, and improper liquefaction of the sputum has been attributed to false-negative results [92].

Timing of the specimen collection after the onset of symptoms: The probability of detection of SARS-CoV-2 from NP/OP swabs by RT-qPCR alone decreases with the time since the onset of symptoms. The longer the time since the onset of symptoms until the case is tested, the more likely chance of false-negative results [93]. In a recent report from John Hopkins, during the first four days of infection prior to the onset of symptoms (day 5), the probability of a false-negative in an individual decreased from 100% on day 1 to 61% on day 4. The probability of a false-negative rate on the day of onset of symptoms (day 5) was 35%. This probability decreased to 26% on day 8 (three days after onset of symptoms) and then started to increase (27%) on day 9 and 61% on day 21 [94].

Difference in severity of infection: Individual differences in patients with regard to the severity of infection, viral load in the upper respiratory tracts may also account for the false negative test results [93].

PCR inhibitors: Common inhibitors in diagnostic PCRs include heme and humic acid. Antiviral drugs like Acyclovir also have been reported to inhibit *Taq* DNA polymerase [95]. Samples from patients with a history of administration of such drugs prior to testing have a higher chance of a false-negative PCR test.

Inexperienced laboratory staff and inadequate infrastructure in laboratories can lead to false positive results due to contamination and cross reactivity.

False-negative antibody ELISA results: The sample used for antibody detection by ELISA is mostly blood. Blood being a uniform sample removes the inconsistency, which can occur with swab collection. However, care should be taken while selecting the collection tube, storage and transportation solutions. Blood being a nutrient-rich substance can get contaminated easily and it calls for ascetic handling while separating plasma or serum. It has been shown that blood samples contaminated with mold or bacteria give higher than normal OD in ELISA. Improper handling and storage can lead to hemolysis of blood. Even though ELISA is relatively immune to artifacts resulting from hemolysis, changes in final OD values have been recorded.

## 5. Conclusions

This review compiles the available test platforms for the diagnosis of COVID-19, one of the most dreaded infections of mankind in the 21st century. The global scientific community is engraved in developing measures to contain this infection using a multitude of methodologies and diagnosis, which is the starting step in the process of controlling the contagion. The diagnosis has diverse magnitudes; at the individual level it deals with the prognosis of the patient, at the population level it provides the epidemiological surveillance data, and at the mass control level it speaks about the herd immunity. The presence of asymptomatic cases with potential to transmit the infection further stresses the importance of accurate diagnosis in real time. The ratio of asymptomatic infections to the symptomatic infection varies from study to study but comes in the range of almost 2–41% [87,88,89]. The spectrum of asymptomatic infections will provide insight into the epidemic spread [87]. Even though the RT–qPCR test of asymptomatic cases proves the potential threat from these patients, no data are available to show the actual threat from these asymptomatic patients [6]. COVID-19 testing rates and testing capacity of the infection is essential for confirming the trajectory of the epidemic [90]. It can be seen that almost all the control strategies begin with the diagnosis data, which speaks of the importance of the accuracy of diagnosis. Even though there are a number of test platforms available from a number of vendors for correctly detecting the virus at different stages of infection, the possibility for false positives and false negatives cannot be over ruled. This review is an attempt to highlight the possible pitfalls in COVID-19 diagnosis to the attention of clinical laboratories.

## Figures and Tables

**Table 1:** List of major commercial Reverse Transcription quantitative Polymerase Chain Reaction (RT-qPCR) assays available for Severe Acute Respiratory Syndrome CoronaVirus 2 (SARS-CoV-2) diagnosis.

No	Test Name	SARS-CoV-2 Target Genes	Control Assay	Assay Type	Sensitivity (Limit of Detection)	Regulatory Status	Company
1	RealStar SARS-CoV-2 RT-PCR Kit 1.0	*E and S*	Internal control	Multiplex	NA	FDA EUA, CE-IVD	Altona Diagnostics GmbH, Hamburg, Germany
2	TaqPath COVID-19 RT-PCR	*ORF1ab*, *N*, *S*	MS2 phage internal control	Multiplex	NA	US FDA EUA, CE-IVD	Applied Biosystems - Thermo Fisher, CA, USA
3	2019-nCoV: Real-Time Fluorescent RT-PCR kit	*ORF1ab*	β-Actin	Multiplex	150 viral copies/mL from thrat swab samples	NMPA Certified/CE Marked/FDA Approved/PMDA Approved	BGI Genomics Co. Ltd., Shenzhen, China
4	SARS-CoV-2 R-GENE test	*N*, *RdRp and E*	Internal control	Multiplex	380 copies/mL	CE-IVD	bioMérieux SA, Marcy-l’Étoile, France
5	CDC 2019-Novel Coronavirus (2019-nCoV) Real-Time RT-PCR Diagnostic Panel	*N1*, *N2*, *RP*	Rnase P	Singleplex	1 to 3.2 copies/μL	FDA EUA	Center for Disease Control, Atlanta, GA, USA
6	EURORealTime SARS-CoV-2	*ORF1ab and N*	internal extraction control	Multiplex	1 copy/μL nucleic acid eluate	CE-IVD	EUROIMMUN AG, Lubeck, Germany
7	SARS-CoV-2 Real-time RT-PCR Assay	*ORF1ab and N*	MS2 phage internal control	Multiplex	20 copies/mL	FDA EUA	PerkinElmer Inc, Austin, TX, USA
8	geneSig- Real Time PCR Coronavirus COVID-19	*RdRp*	internal extraction control	Multiplex	0.58 copies/μL of SARS-CoV-2 viral RNA	US FDA EUA - CE-IVD - WHO EUL	Primerdesign Ltd., Chandler’s Ford, UK
9	Lyra SARS-CoV-2 Assay	*Nsp pp1ab*	MS2 phage internal control	Multiplex	8.00E-01 genomic RNA copies/μL	FDA EUA, CE	Quidel Corporation, San Diego, CA, USA
10	cobas SARS-CoV-2 Test	*ORF-1a/b* & *E*	MS2 phage internal control	Using cobas 6800 system, multiplex	689.3 copies/mL	FDA EUA, CE-IVD	Roche Diagnostics Mannheim, Germany
11	Allplex 2019-nCoV Assay	*E*, *RdRP* & *N*	MS2 phage control	Multiplex	1250 to 4167 copies/mL	US, FDA EUA, - Korea MFDS EUA - Singapore HSA - CE-IVD	Seegene Inc, Seoul, Republic of Korea
12	SARS-CoV-2 RealTime PCR Kit	*N* & *E*	Rnase P	Multiplex	50 copies/tube	CE-IVD	Vircell SL, Granada, Spain

**Table 2:** Immunological tests available globally for detection of COVID-19.

**1. ELISAs Including Automated Immunoassays (IAs)**									
**No**	**Name of Test**	**Assay Type**	**Capture Antigen**	**Target Analyte**	**Specificity**	**Sensitivity**	**Regulatory Status**	**Assay Read out**	**Company/Location**
1	Wantai SARS-CoV-2 IgM ELISA	antibody capture ELISA	RBD domain	IgM	100%	86.8%	CE-IVD	Microplate reader	Beijing Wantai Biological Pharmacy Enterprise Co., Ltd.
2	Wantai SARS-CoV-2 Ab ELISA	double antigen	RBD domain	Total Ab	100%	94.5%	CE-IVD	Microplate reader	Beijing Wantai Biological Pharmacy Enterprise Co., Ltd.
3	EDI Novel Coronavirus COVID-19 IgG ELISA kit	indirect ELISA	Not provided	IgG	Not provided	Not provided	CE-IVD	Microplate reader	Epitope Diagnostics, Inc.
4	EDI Novel Coronavirus COVID-19 IgM ELISA kit	indirect ELISA	Not provided	IgM	Not provided	Not provided	CE-IVD	Microplate reader	Epitope Diagnostics, Inc.
5	Anti-SARS-CoV-2 ELISA (IgA)	indirect ELISA	S1 with RBD region	IgA	92%	98.6%	CE-IVD	Microplate reader	EUROIMMUN AG
6	Anti-SARS-CoV-2 ELISA (IgG)	indirect ELISA	S1 domain of spike	IgG	99.6%	94.4%	CE-IVD; USA; Brazil	Microplate reader	EUROIMMUN AG
7	2019 Novel Coronavirus IgG Test (ELISA)	indirect ELISA	Not provided	IgG	Not provided	Not provided	RUO	Microplate reader	Guangzhou Darui Biotechnology Co., Ltd.
8	2019 Novel Coronavirus IgM Test (ELISA)	indirect ELISA	Not provided	IgM	Not provided	Not provided	RUO	Microplate reader	Guangzhou Darui Biotechnology Co., Ltd.
9	VITROS^®^ SARS-CoV-2 Antibody kit	micro well, Chemiluminescence	Not provided	IgG	100%	90%	RUO	VITROS ECi/ECiQ/3600	Ortho-Clinical Diagnostics, Inc
10	COVID-19 ELISA IgG Antibody Test	Indirect ELISA	Not provided	IgG	100%	92.5%	EUA 4/15/2020	Microplate reader	Mount Sinai Laboratory
11	LIAISON^®^ SARS-CoV-2 IgG | DiaSorin	Magnetic beads Chemiluminescence	S1 and S2 spike domain	IgG	97.4	98.5	RUO	Dedicated equipment	DiaSorin Inc.
12	Platelia SARS-CoV-2 Total Ab Assay	indirect ELISA	nucleoprotein	All Ig	99.56	100	EUA 4/29/2020	Microplate reader	Bio-Rad Co., Ltd.
13	Elecsys anti-SARS-CoV-2 serology test	Electro-chemiluminescence immunoassay (ECLIA)	Not provided	IgG	99.8	100	RUO	cobas e analyser series	Roche Co., Ltd.
14	New York SARS-CoV Microsphere Immunoassay	luminex	nucleocapsid of SARS-CoV-1	IgG	79.3	96.7	RUO	Luminex reader	Wadsworth Center, New York State Department of Health
15	Abbott Alinity i ARS-CoV-2 IgG	Chemilumunscnece	Nucleocapsid	IgG	100	99.9	EUA 05/11/2020	ARCHITECT and Alinity systems	Abbott Laboratories
16	Diazyme DZ-LITE SARS-CoV-2 IgG, IgM CLIA Kits	chemiluminescence	Not provided	IgG/IgM	97.3	91.2	Not provided	Not provided	Diazyme Laboratories
17	NovaLisa^®^ SARS-CoV-2 IgG	indirect ELISA	Not provided	IgG	Not provided	Not provided	CE-IVD CE mark 4/2020	Microplate reader	Gold Standard Diagnostics/Eurofins Technologies
18	ErbaLisa COVID-19 ELISA kits	indirect ELISA	Not provided	IgG	98.1	98.3	CE mark 4/2020	Microplate reader	Erba Mannheim
19	UBI SARS-CoV-2 ELISA	indirect ELISA	Not provided		NA	NA	CE mark 4/2020	Microplate reader	United Biomedical
**2. Antigen (Ag)-based LFAs**									
	**Name of Test**	**Assay Type**	**Capture Antibody**	**Target analyte**	**Specificity**	**Sensitivity**	**Regulatory status**	**Assay Read out**	**Company/location**
1	COVID-19 Ag Respi-Strip	gold conjugate	Not provided	Not provided	100	60	CE-IVD	Visual	Coris BioConcept
2	BIOCREDIT COVID-19 Ag	gold conjugate	Not provided	Not provided	Not provided	Not provided	CE-IVD	Visual	RapiGEN, Inc.
3	STANDARD F COVID-19 Ag FIA	time-resolved fluorescence europium	Not provided	Not provided	Not provided	Not provided	CE-IVD	Reader	SD BIOSENSOR, INC.
4	STANDARD Q COVID-19 Ag Test	gold conjugate	Not provided	Not provided	Not provided	Not provided	CE-IVD	Visual	SD BIOSENSOR, INC.
5	BIOEASY 2019-nCoV Ag Fluorescence Rapid Test Kit (time-resolved fluorescence)	time-resolved fluorescence	Not provided	Not provided	Not provided	Not provided	CE-IVD	Reader	Shenzhen Bioeasy Biotechnology Co., Ltd.
6	Sofia 2 SARS Antigen FIA	time-resolved fluorescence	Not provided	Not provided	Not provided	Not provided	EUA 5/8/2020	Reader	Quidel Co., Ltd.
7	ichromaTM COVID-19 Ag test	time-resolved fluorescence	Not provided	Not provided	97	95.8	CE-IVD	Reader	Boditech Co., Ltd.
8	2019-Novel Coronavirus (2019-nCoV) Antigen Rapid Test Kit (FIA)	fluorescence	Not provided	Not provided	Not provided	Not provided	CE-IVD	Reader	Bioeasy Co., Ltd.
**3. Antibody based LFAs**									
**No**	**Name of Test**	**Assay Type**	**Capture Antibody**	**Target analyte**	**Specificity**	**Sensitivity**	**Regulatory status**	**Assay Read out**	**Company/location**
1	2019-nCoV IgG/IgM Antibody Determination Kit	gold conjugate	Not provided	IgM/IgG	NA	NA	CE-IVD	Reader required	Beijing Diagreat Biotechnologies Co., Ltd.
2	Tigsun COVID-19 Combo IgM/IgG Rapid Test (lateral flow)	gold conjugate	Not provided	IgM/IgG	NA	NA	CE-IVD; India	Visual	Beijing Tigsun Diagnostics Co., Ltd.
3	Wantai SARS-CoV-2 Ab Rapid Test	gold conjugate	Not provided	Total Ab	NA	NA	Australia	Visual	Beijing Wantai Biological Pharmacy Enterprise Co., Ltd.
4	COVID-19 IgM-IgG Combined Antibody Rapid Test	gold conjugate	Not provided	IgM/IgG	NA	NA	CE-IVD; India	Visual	BioMedomics, Inc.
5	iChroma COVID-19 Ab	time-resolved fluorescence	Not provided	IgM/IgG	96.7	95.8	RUO	Reader required	Boditech Inc.
6	Rapid Response COVID-19 IgG/IgM Test Cassette (whole blood/serum/plasma)	gold conjugate	Not provided	IgM/IgG	NA	NA	RUO	Visual	BTNX, Inc.
7	Cellex qSARS-CoV-2 IgG/IgM Cassette Rapid Test	gold conjugate	Not provided	IgM/IgG	NA	NA	CE-IVD; USA	Visual	Cellex, Inc.
8	COVID-19 IgM/IgG Ab Test	gold conjugate	Not provided	IgM/IgG	NA	NA	CE-IVD	Visual	Core Technology Co., Ltd.
9	2019-nCoV IgG/IgM Rapid Test	gold conjugate	Not provided	IgM/IgG	92	NA	CE-IVD	Visual	Dynamiker Biotechnology Co., Ltd.
10	GenBody COVID-19 IgM/IgG	gold conjugate	Not provided	IgM/IgG	97.5	95.2	CE-IVD; Australia; Brazil	95.2	GenBody Inc.
11	RightSign COVID-19 IgG/IgM Rapid Test	gold conjugate	Not provided	IgM/IgG	NA	NA	CE-IVD	Visual	Hangzhou Biotest Biotech Co., Ltd.
12	PerfectPOC Novel Corona Virus (SARS-CoV-2) IgM/IgG Rapid Test Kit	gold conjugate	Not provided	IgM/IgG	95.7	NA	CE-IVD	Visual	Jiangsu Bioperfectus Technology Co., Ltd.
13	HIGHTOP COVID-19 IgM/IgG Ab Rapid Test Kit	gold conjugate	Not provided	IgM/IgG	NA	NA	CE-IVD	Visual	Qingdao Hightop Biotech Co., Ltd.
14	BIOCREDIT COVID-19 IgG+IgM Duo	gold conjugate	Not provided	IgM/IgG	NA	NA	CE-IVD	Visual	RapiGEN Co., Ltd.
15	STANDARDTM Q COVID-19 IgM/IgG Combo Test	gold conjugate	Not provided	IgM/IgG	95.09	94.3	CE-IVD; Brazil	Visual	SD BIOSENSOR, INC
16	BIOEASY 2019-nCoV Ab (IgG/IgM) GICA Rapid Test Kit	gold conjugate	Not provided	IgM/IgG	NA	NA	CE-IVD	Visual	Bioeasy Biotechnology Co., Ltd.
17	VivaDiagTM COVID-19 IgM/IgG Rapid Test	gold conjugate	Not provided	IgM/IgG	NA	NA	CE-IVD	Visual	VivaChek Biotech (Hangzhou) Co., Ltd.
18	Diagnostic Kit for IgG Antibody to Corona Virus (nCoV-2019)	gold conjugate	Not provided	IgG + IgM	NA	NA	CE-IVD; China	Visual	Zhuhai Livzon Diagnostics, Inc.
19	COVID-19 IgG/IgM Rapid Test	gold conjugate	Not provided	IgG + IgM	NA	NA	CE-IVD	Visual	Assure Tech Co., Ltd.
20	Novel Coronavirus IgM/IgG Combo Rapid Test	gold conjugate	Not provided	IgG + IgM	NA	NA	EUA submission pending	Visual	Decombio Biotechnology Co., Ltd.
21	SARS-CoV-2 IgG/IgM Antibody Detection Kit	gold conjugate	Not provided	IgG + IgM	NA	NA	CE mark 4/2020	Visual	Beroni Group Co., Ltd.
22	2019-nCoV IgG/IgM Detection Kit (Colloidal Gold)	gold conjugate	Not provided	IgG + IgM	NA	NA	CE mark 4/2020	Visual	Biolidics Co., Ltd.
23	COVID-19 IgM-IgG Rapid Test	gold conjugate	Not provided	IgG + IgM	NA	NA	EUA submission pending	Visual	BioMedomics Co., Ltd.
24	AccuRapid SARS-CoV-2 IgM/IgG Test Kit (Lateral Flow Immunoassay	gold conjugate	Not provided	IgG + IgM	NA	NA	EUA submission pending	Visual	Eachy Biopharmaceuticals Co., Ltd.
25	One Step SARS-CoV-2 (COVID-19) IgG/IgM Test	gold conjugate	Not provided	IgG + IgM	NA	NA	CE mark 5/2020	Visual	Hangzhou Testsea biotechnology Co., LTD
26	COVID-19 IgG/IgM Rapid Test	gold conjugate	Not provided	IgG + IgM	97.5	96.7	CE mark 5/2020	Visual	Healgen Scientific, LLC
27	SARS-CoV-2 IgM/IgG Antibody Rapid Test Kit	gold conjugate	Not provided	IgG + IgM	98.7	93.3	CE mark 5/2020	Visual	Nanjing Liming Bio-products Co., Ltd.
28	COVID-19 (SARS-CoV-2) IgG Antibody Detection Kit	gold conjugate	Not provided	IgG + IgM	100	97.5	CE mark 5/2020	Visual	Nirmidas Biotech Co., Ltd.
29	PCL COVID-19 IgG/IgM Rapid Gold	gold conjugate	Not provided	IgG+ IgM	NA	NA	CE mark 5/2020	Visual	Vitrex Medical A/S
30	MosaiQ COVID-19 Antibody Microarray	fluorescence	Not provided	IgG/IgM	99.8	100	CE mark 5/2020	Reader	Quotient Limited Co., Ltd.
31	SureScreen COVID-19 IgM/IgG Rapid Test Cassette	gold conjugate	Not provided	IgG+ IgM	99	91	CE mark 2020	Visual	SureScreen Diagnostics Co., Ltd.
32	SARS-CoV-2 IgG/IgM Antibody Detection Kit	gold conjugate	Not provided	IgG+ IgM	NA	NA	CE mark 2020	Visual	Tianjin Beroni Biotechnology Co., Ltd.
33	Diagnostic Kit for IgM/IgG Antibody to Coronavirus (SARS-CoV-2)	gold conjugate	Not provided	IgG+ IgM	NA	NA	CE mark 2020	Visual	Zhuhai Livzon Diagnostics Co., Ltd.

CE-IVD—Certificate in-vitro Diagnostics; RUA—Research Use Only; EUA—Emergency Use Authorization.

## References

[1] Wu F., Zhao S., Yu B., Chen Y.M., Wang W., Song Z.G., Hu Y., Tao Z.W., Tian J.H., Pei Y.Y. (2020). A new coronavirus associated with human respiratory disease in China. Nature.

[2] Zhu N., Zhang D., Wang W., Li X., Yang B., Song J., Zhao X., Huang B., Shi W., Lu R. (2020). A novel coronavirus from patients with pneumonia in China, 2019. N. Engl. J. Med..

[3] Fehr A.R., Perlman S. (2015). Coronaviruses: An overview of their replication and pathogenesis. Coronaviruses: Methods and Protocols.

[4] Dhama K., Khan S., Tiwari R., Sircar S., Bhat S., Malik Y.S., Singh K.P., Chaicumpa W., Bonilla-Aldana D.K., Rodriguez-Morales A.J. (2020). Coronavirus disease 2019–COVID-19. Clin. Microbiol. Rev..

[5] Cucinotta D., Vanelli M. (2020). WHO declares COVID-19 a pandemic. Acta Biomed..

[6] Wölfel R., Corman V.M., Guggemos W., Seilmaier M., Zange S., Müller M.A., Niemeyer D., Jones T.C., Vollmar P., Rothe C. (2020). Virological assessment of hospitalized patients with COVID-2019. Nature.

[7] Corman V., Bleicker T., Brunink S., Drosten C. (2020). Diagnostic Detection of Wuhan Coronavirus 2019 by Real-Time RTPCR. https://www.who.int/docs/default-source/coronaviruse/wuhan-virus-assay-v1991527e5122341d99287a1b17c111902.pdf.

[8] Wang W., Xu Y., Gao R., Lu R., Han K., Wu G., Tan W. (2020). Detection of SARS-CoV-2 in Different Types of Clinical Specimens. JAMA J. Am. Med. Assoc..

[9] Pan Y., Zhang D., Yang P., Poon L.L.M., Wang Q. (2020). Viral load of SARS-CoV-2 in clinical samples. Lancet Infect. Dis..

[10] See A., Toh S.T. (2020). Respiratory sampling for severe acute respiratory syndrome coronavirus 2: An Overview. Head Neck.

[11] Pan D., Sze S., Rogers B., Bron J., Bird P.W., Holmes C.W., Tang J.W. (2020). Serial simultaneously self-swabbed samples from multiple sites show similarly decreasing SARS-CoV-2 loads in COVID-19 cases of differing clinical severity. J. Infect..

[12] Loeffelholz M.J., Tang Y.W. (2020). Laboratory diagnosis of emerging human coronavirus infections–the state of the art. Emerg. Microbes Infect..

[13] To K.K.-W., Tsang O.T.-Y., Leung W.-S., Tam A.R., Wu T.-C., Lung D.C., Yip C.C.-Y., Cai J.-P., Chan J.M.-C., Chik T.S.-H. (2020). Temporal profiles of viral load in posterior oropharyngeal saliva samples and serum antibody responses during infection by SARS-CoV-2: An observational cohort study. Lancet Infect. Dis..

[14] Leung E.C., Chow V.C., Lee M.K., Lai R.W. (2020). Deep throat saliva as an alternative diagnostic specimen type for the detection of SARS-CoV-2. J. Med. Virol..

[15] Lai C.K.C., Chen Z., Lui G., Ling L., Li T., Wong M.C.S., Ng R.W.Y., Tso E.Y.K., Ho T., Fung K.S.C. (2020). Prospective Study Comparing Deep Throat Saliva With Other Respiratory Tract Specimens in the Diagnosis of Novel Coronavirus Disease 2019. J. Infect. Dis..

[16] Kojima N., Turner F., Slepnev V., Bacelar A., Deming L., Kodeboyina S., Klausner J.D. (2020). Self-Collected Oral Fluid and Nasal Swabs Demonstrate Comparable Sensitivity to Clinician Collected Nasopharyngeal Swabs for Covid-19 Detection. medRxiv.

[17] Wyllie A.L., Fournier J., Casanovas-Massana A., Campbell M., Tokuyama M., Vijayakumar P., Warren J.L., Geng B., Muenker M.C., Moore A.J. (2020). Saliva or Nasopharyngeal Swab Specimens for Detection of SARS-CoV-2. N. Engl. J. Med..

[18] Abdollahi A., Shakoori A., Khoshnevis H., Arabzadeh M., Manshadi S.A.D., Mohammadnejad E., Ghasemi D., Aboksari M.S., Alizadeh S., Mehrtash V. (2020). Comparison of patient-collected and lab technician-collected nasopharyngeal and oropharyngeal swabs for detection of COVID-19 by RT-PCR. Iran. J. Pathol..

[19] Guest J.L., Sullivan P.S., Valentine-Graves M., Valencia R., Adam E., Luisi N., Nakano M., Guarner J., del Rio C., Sailey C. (2020). Suitability and Sufficiency of Telehealth Clinician-Observed, Participant-Collected Samples for SARS-CoV-2 Testing: The iCollect Cohort Pilot Study. JMIR Public Health Surveill..

[20] Yang Y., Yang M., Shen C., Wang F., Yuan J., Li J., Zhang M., Wang Z., Xing L., Wei J. (2020). Evaluating the accuracy of different respiratory specimens in the laboratory diagnosis and monitoring the viral shedding of 2019-nCoV infections. medRxiv.

[21] CDC Interim Guidelines for Collecting, Handling, and Testing Clinical Specimens from Persons for Coronavirus Disease 2019 (COVID-19). https://www.cdc.gov/coronavirus/2019-ncov/lab/guidelines-clinical-specimens.html.

[22] Karthik K., Aravindh Babu R.P., Dhama K., Chitra M.A., Kalaiselvi G., Alagesan Senthilkumar T.M., Raj G.D. (2020). Biosafety Concerns During the Collection, Transportation, and Processing of COVID-19 Samples for Diagnosis. Arch. Med. Res..

[23] Yu F., Qiu T., Zeng Y., Wang Y., Zheng S., Chen X., Chen Y. (2018). Comparative evaluation of three preprocessing methods for extraction and detection of influenza A virus nucleic acids from sputum. Front. Med..

[24] Sung H., Yong D., Ki C.S., Kim J.S., Seong M.W., Lee H., Kim M.N. (2016). Comparative evaluation of three homogenization methods for isolating middle east respiratory syndrome coronavirus nucleic acids from sputum samples for real-time reverse transcription PCR. Ann. Lab. Med..

[25] Bruce E.A., Huang M., Perchetti G.A., Tighe S., Laaguiby P., Hoffman J.J., Gerrard D.L., Nalla A.K., Wei Y., Greninger A.L. (2020). Direct RT-qPCR detection of SARS-CoV-2 RNA from patient nasopharyngeal swabs without an rna extraction step. bioRxiv Prepr..

[26] Lübke N., Senff T., Scherger S., Hauka S., Andrée M., Adams O., Timm J., Walker A. (2020). Extraction-free SARS-CoV-2 detection by rapid RT-qPCR universal for all primary respiratory materials. J. Clin. Virol..

[27] D’Cruz R.J., Currier A.W., Sampson V.B. (2020). Laboratory Testing Methods for Novel Severe Acute Respiratory Syndrome-Coronavirus-2 (SARS-CoV-2). Front. Cell Dev. Biol..

[28] Wilkinson D.A. (1998). Getting The Message with RT-PCR. Scientist.

[29] Bustin S.A., Nolan T. (2004). Pitfalls of quantitative real- time reverse-transcription polymerase chain reaction. J. Biomol. Tech..

[30] Corman V.M., Landt O., Kaiser M., Molenkamp R., Meijer A., Chu D.K.W., Bleicker T., Brünink S., Schneider J., Schmidt M.L. (2020). Detection of 2019 novel coronavirus (2019-nCoV) by real-time RT-PCR. Eurosurveillance.

[31] Cheng M.P., Papenburg J., Desjardins M., Kanjilal S., Quach C., Libman M., Dittrich S., Yansouni C.P. (2020). Diagnostic Testing for Severe Acute Respiratory Syndrome-Related Coronavirus-2: A Narrative Review. Ann. Intern. Med..

[32] Vogels C.B.F., Brito A.F., Wyllie A.L., Fauver J.R., Ott I.M., Kalinich C.C., Petrone M.E., Landry M.-L., Foxman E.F., Grubaugh N.D. (2020). Analytical sensitivity and efficiency comparisons of SARS-COV-2 qRT-PCR assays. medRxiv.

[33] Nalla A.K., Casto A.M., Huang M.-L.W., Perchetti G.A., Sampoleo R., Shrestha L., Wei Y., Zhu H., Jerome K.R., Greninger A.L. (2020). Comparative Performance of SARS-CoV-2 Detection Assays using Seven Different Primer/Probe Sets and One Assay Kit. J. Clin. Microbiol..

[34] Kim S., Kim D.-M., Lee B. (2020). Insufficient Sensitivity of RNA Dependent RNA Polymerase Gene of SARS-CoV-2 Viral Genome as Confirmatory Test using Korean COVID-19 Cases. Preprints.

[35] CDC (2020). CDC 2019-Novel Coronavirus (2019-nCoV) Real-Time RT-PCR Diagnostic Panel.

[36] Notomi T., Okayama H., Masubuchi H., Yonekawa T., Watanabe K., Amino N., Hase T. (2000). Loop-mediated isothermal amplification of DNA. Nucleic Acids Res..

[37] Lee S.H., Baek Y.H., Kim Y.H., Choi Y.K., Song M.S., Ahn J.Y. (2017). One-pot reverse transcriptional loop-mediated isothermal amplification (RT-LAMP) for detecting MERS-CoV. Front. Microbiol..

[38] Yan C., Cui J., Huang L., Du B., Chen L., Xue G., Li S., Zhang W., Zhao L., Sun Y. (2020). Rapid and visual detection of 2019 novel coronavirus (SARS-CoV-2) by a reverse transcription loop-mediated isothermal amplification assay. Clin. Microbiol. Infect..

[39] Baek Y.H., Um J., Antigua K.J.C., Park J.-H., Kim Y., Oh S., Kim Y., Choi W.-S., Kim S.G., Jeong J.H. (2020). Development of a reverse transcription-loop-mediated isothermal amplification as a rapid early-detection method for novel SARS-CoV-2. Emerg. Microbes Infect..

[40] Yu L., Wu S., Hao X., Dong X., Mao L., Pelechano V., Chen W.-H., Yin X. (2020). Rapid detection of COVID-19 coronavirus using a reverse transcriptional loop-mediated isothermal amplification (RT-LAMP) diagnostic platform. Clin. Chem..

[41] James P., Stoddart D., Harrington E.D., Beaulaurier J., Ly L., Reid S.W., Turner D.J., Juul S. (2020). LamPORE: Rapid, accurate and highly scalable molecular screening for SARS-CoV-2 infection, based on nanopore sequencing. medRxiv Prepr..

[42] Jinek M., Chylinski K., Fonfara I., Hauer M., Doudna J.A., Charpentier E. (2012). A programmable dual-RNA-guided DNA endonuclease in adaptive bacterial immunity. Science.

[43] Myhrvold C., Freije C.A., Gootenberg J.S., Abudayyeh O.O., Metsky H.C., Durbin A.F., Kellner M.J., Tan A.L., Paul L.M., Parham L.A. (2018). Field-deployable viral diagnostics using CRISPR-Cas13. Science.

[44] Joung J., Adha A., Segel M., Li J., Walker B.D., Greninger A.L., Jerome K.R. (2020). Point-of-care testing for COVID-19 using SHERLOCK diagnostics. medRxiv.

[45] Chen J.S., Ma E., Harrington L.B., Da Costa M., Tian X., Palefsky J.M., Doudna J.A. (2018). CRISPR-Cas12a target binding unleashes indiscriminate single-stranded DNase activity. Science.

[46] Broughton J.P., Deng X., Yu G., Fasching C.L., Servellita V., Singh J., Miao X., Streithorst J.A., Granados A., Sotomayor-gonzalez A. (2020). CRISPR—Cas12-based detection of SARS-CoV-2. Nat. Biotechnol..

[47] Acharya S., Mishra A., Paul D., Ansari A.H., Azhar M., Kumar M., Rauthan R., Sharma N., Aich M., Sinha D. (2019). Francisella novicida Cas9 interrogates genomic DNA with very high specificity and can be used for mammalian genome editing. Proc. Natl. Acad. Sci. USA.

[48] Azhar M., Phutela R., Ansari A.H., Sinha D., Sharma N., Kumar M., Aich M., Sharma S., Rauthan R., Singhal K. (2020). Rapid, field-deployable nucleobase detection and identification using FnCas9. bioRxiv.

[49] Ackerman C.M., Myhrvold C., Thakku S.G., Freije C.A., Metsky H.C., Yang D.K., Ye S.H., Boehm C.K., Kosoko-Thoroddsen T.-S.F., Kehe J. (2020). Massively multiplexed nucleic acid detection using Cas13. Nature.

[50] Nikam C., Kazi M., Nair C., Jaggannath M., Manoj M.M., Vinaya R.V., Shetty A., Rodrigues C. (2014). Evaluation of the Indian TrueNAT micro RT-PCR device with GeneXpert for case detection of pulmonary tuberculosis. Int. J. Mycobacteriol..

[51] Natesan S., Bhatia R., Sundararajan A., Dhama K., Malik Y.S., Vora K. (2020). Ramping up of SARS CoV-2 testing for the diagnosis of COVID-19 to better manage the next phase of pandemic and reduce the mortality in India. VirusDisease.

[52] Yelin I., Aharony N., Shaer-Tamar E., Argoetti A., Messer E., Berenbaum D., Shafran E., Kuzli A., Gandali N., Hashimshony T. (2020). Evaluation of COVID-19 RT-qPCR test in multi-sample pools. medRxiv.

[53] Lohse S., Pfuhl T., Berkó-Göttel B., Rissland J., Geißler T., Gärtner B., Becker S.L., Schneitler S., Smola S. (2020). Pooling of samples for testing for SARS-CoV-2 in asymptomatic people. Lancet Infect. Dis..

[54] Ben-Ami R., Klochendler A., Seidel M., Sido T., Gurel-Gurevich O., Yassour M., Meshorer E., Benedek G., Fogel I., Oiknine-Djian E. (2020). Large-scale implementation of pooled RNA extraction and RT-PCR for SARS-CoV-2 detection. Clin. Microbiol. Infect..

[55] Abdalhamid B., Bilder C.R., McCutchen E.L., Hinrichs S.H., Koepsell S.A., Iwen P.C. (2020). Assessment of Specimen Pooling to Conserve SARS CoV-2 Testing Resources. Am. J. Clin. Pathol..

[56] Ben-Shmuel A., Brosh-Nissimov T., Glinert I., Bar-David E., Sittner A., Poni R., Cohen R., Achdout H., Tamir H., Yahalom-Ronen Y. (2020). Detection and infectivity potential of Severe Acute Respiratory Syndrome Coronavirus 2 (SARS-CoV-2) environmental contamination in isolation units and quarantine facilities. Clin. Microbiol. Infect..

[57] Ding Z., Qian H., Xu B., Huang Y., Miao T., Yen H.L., Xiao S., Cui L., Wu X., Shao W. (2020). Toilets dominate environmental detection of severe acute respiratory syndrome coronavirus 2 in a hospital. Sci. Total Environ..

[58] Orive G., Lertxundi U., Barcelo D. (2020). Early SARS-CoV-2 outbreak detection by sewage-based epidemiology. Sci. Total Environ..

[59] WHO (2020). “Immunity Passports” in the Context of COVID-19.

[60] Seo G., Lee G., Kim M.J., Baek S.-H., Choi M., Ku K.B., Lee C.-S., Jun S., Park D., Kim H.G. (2020). Rapid Detection of COVID-19 Causative Virus (SARS-CoV-2) in Human Nasopharyngeal Swab Specimens Using Field-Effect Transistor-Based Biosensor. ACS Nano.

[61] Nag P., Sadani K., Mukherji S. (2020). Optical Fiber Sensors for Rapid Screening of COVID-19. Trans. Indian Natl. Acad. Eng..

[62] Murugan D., Bhatia H., Sai V.V.R., Satija J. (2020). P-FAB: A Fiber-Optic Biosensor Device for Rapid Detection of COVID-19. Trans. Indian Natl. Acad. Eng..

[63] Tripathi S., Agrawal A. (2020). Blood Plasma Microfluidic Device: Aiming for the Detection of COVID-19 Antibodies Using an On-Chip ELISA Platform. Trans. Indian Natl. Acad. Eng..

[64] Jääskeläinen A.J., Kekäläinen E., Kallio-Kokko H., Mannonen L., Kortela E., Vapalahti O., Kurkela S., Lappalainen M. (2020). Evaluation of commercial and automated SARS-CoV-2 IgG and IgA ELISAs using coronavirus disease (COVID-19) patient samples. Eurosurveillance.

[65] Adams E.R., Anand R., Andersson M.I., Auckland K., Baillie J.K., Barnes E., Bell J., Berry T., Bibi S., Carroll M. (2020). Evaluation of antibody testing for SARS-Cov-2 using ELISA and lateral flow immunoassays. medRxiv.

[66] Graham D.A., Mawhinney K.A., McShane J., Connor T.J., Adair B.M., Merza M. (1997). Standardization of enzyme-linked immunosorbent assays (ELISAs) for quantitative estimation of antibodies specific for infectious bovine rhinotracheitis virus, respiratory syncytial virus, parainfluenza-3 virus, and bovine viral diarrhea virus. J. Vet. Diagn. Investig..

[67] Chen S., Lu D., Zhang M., Che J., Yin Z., Zhang S., Zhang W., Bo X., Ding Y., Wang S. (2005). Double-antigen sandwich ELISA for detection of antibodies to SARS-associated coronavirus in human serum. Eur. J. Clin. Microbiol. Infect. Dis..

[68] Zhao J., Yuan Q., Wang H., Liu W., Liao X., Su Y., Wang X., Yuan J., Li T., Li J. (2020). Antibody responses to SARS-CoV-2 in patients of novel coronavirus disease 2019. Clin. Infect. Dis..

[69] Lin D., Liu L., Zhang M., Hu Y., Yang Q., Guo J., Dai Y., Xu Y., Cai Y., Chen X. (2020). Evaluations of serological test in the diagnosis of 2019 novel coronavirus (SARS-CoV-2) infections during the COVID-19 outbreak. medRxiv.

[70] Vashist S.K. (2020). In vitro diagnostic assays for COVID-19: Recent advances and emerging trends. Diagnostics.

[71] Taylor J. New York SARS-CoV Microsphere Immunoassay for Antibody Detection. https://www.fda.gov/media/137540/download.

[72] Kimpston-Burkgren K., Mora-Díaz J.C., Roby P., Bjustrom-Kraft J., Main R., Bosse R., Giménez-Lirola L.G. (2020). Characterization of the humoral immune response to porcine epidemic diarrhea virus infection under experimental and field conditions using an AlphaLISA platform. Pathogens.

[73] Perera R.A., Mok C.K., Tsang O.T., Lv H., Ko R.L., Wu N.C., Yuan M., Leung W.S., Chan J.M., Chik T.S. (2020). Serological assays for severe acute respiratory syndrome coronavirus 2 (SARS-CoV-2), March 2020. Eurosurveillance.

[74] Vlasova A.N., Zhang X., Hasoksuz M., Nagesha H.S., Haynes L.M., Fang Y., Lu S., Saif L.J. (2007). Two-Way Antigenic Cross-Reactivity between Severe Acute Respiratory Syndrome Coronavirus (SARS-CoV) and Group 1 Animal CoVs Is Mediated through an Antigenic Site in the N-Terminal Region of the SARS-CoV Nucleoprotein. J. Virol..

[75] Yong S.E.F., Anderson D.E., Wei W.E., Pang J., Chia W.N., Tan C.W., Teoh Y.L., Rajendram P., Toh M.P.H.S., Poh C. (2020). Connecting clusters of COVID-19: An epidemiological and serological investigation. Lancet Infect. Dis..

[76] Koczula K.M., Gallotta A. (2016). Lateral flow assays. Essays Biochem..

[77] Sajid M., Kawde A.N., Daud M. (2015). Designs, formats and applications of lateral flow assay: A literature review. J. Saudi Chem. Soc..

[78] Bendavid E., Mulaney B., Sood N., Shah S., Ling E., Bromley-Dulfano R., Lai C., Weissberg Z., Saavedra R., Tedrow J. (2020). COVID-19 Antibody Seroprevalence in Santa Clara County, California. medRxiv.

[79] Lisboa Bastos M., Tavaziva G., Abidi S.K., Campbell J.R., Haraoui L.-P., Johnston J.C., Lan Z., Law S., MacLean E., Trajman A. (2020). Diagnostic accuracy of serological tests for covid-19: Systematic review and meta-analysis. BMJ.

[80] Sun B., Feng Y., Mo X., Zheng P., Wang Q., Li P., Peng P., Liu X., Chen Z., Huang H. (2020). Kinetics of SARS-CoV-2 specific IgM and IgG responses in COVID-19 patients. Emerg. Microbes Infect..

[81] Krüttgen A., Cornelissen C.G., Dreher M., Hornef M., Imöhl M., Kleines M. (2020). Comparison of four new commercial serologic assays for determination of SARS-CoV-2 IgG. J. Clin. Virol..

[82] Montesinos I., Gruson D., Kabamba B., Dahma H., Van den Wijngaert S., Reza S., Carbone V., Vandenberg O., Gulbis B., Wolff F. (2020). Evaluation of two automated and three rapid lateral flow immunoassays for the detection of anti-SARS-CoV-2 antibodies. J. Clin. Virol..

[83] Liu Y., Liu Y., Diao B., Ren F., Wang Y., Ding J., Huang Q. (2020). Diagnostic Indexes of a Rapid IgG/IgM Combined Antibody Test for SARS-CoV-2. medRxiv.

[84] Lee N.Y., Li C.W., Tsai H.P., Chen P.L., Syue L.S., Li M.C., Tsai C.S., Lo C.L., Hsueh P.R., Ko W.C. (2020). A case of COVID-19 and pneumonia returning from Macau in Taiwan: Clinical course and anti-SARS-CoV-2 IgG dynamic. J. Microbiol. Immunol. Infect..

[85] Gorse G.J., Donovan M.M., Patel G.B. (2020). Antibodies to coronaviruses are higher in older compared with younger adults and binding antibodies are more sensitive than neutralizing antibodies in identifying coronavirus-associated illnesses. J. Med. Virol..

[86] Okba N.M.A., Müller M.A., Li W., Wang C., GeurtsvanKessel C.H., Corman V.M., Lamers M.M., Sikkema R.S., de Bruin E., Chandler F.D. (2020). Severe Acute Respiratory Syndrome Coronavirus 2-Specific Antibody Responses in Coronavirus Disease 2019 Patients. Emerg. Infect. Dis..

[87] Nishiura H., Jung S., Linton N.M., Kinoshita R., Yang Y., Hayashi K., Kobayashi T., Yuan B., Akhmetzhanov A.R. (2020). The Extent of Transmission of Novel Coronavirus in Wuhan, China, 2020. J. Clin. Med..

[88] Ma Y., Xu Q.N., Wang F.L., Ma X.M., Wang X.Y., Zhang X.G., Zhang Z.F. (2020). Characteristics of asymptomatic patients with SARS-CoV-2 infection in Jinan, China. Microbes Infect..

[89] Mizumoto K., Chowell G. (2020). Transmission potential of the novel coronavirus (COVID-19) onboard the diamond Princess Cruises Ship, 2020. Infect. Dis. Model..

[90] Omori R., Mizumoto K., Chowell G. (2020). Changes in testing rates could mask the novel coronavirus disease (COVID-19) growth rate. Int. J. Infect. Dis..

[91] Hong K.H., Lee S.W., Kim T.S., Huh H.J., Lee J., Kim S.Y., Park J.-S., Kim G.J., Sung H., Roh K.H. (2020). Guidelines for Laboratory Diagnosis of Coronavirus Disease 2019 (COVID-19) in Korea. Ann. Lab. Med..

[92] Xu H., Yan L., Qiu C.M., Jiao B., Chen Y., Tan X., Chen Z., Ai L., Xiao Y., Luo A. (2020). Analysis and Prediction of False Negative Results for SARS-CoV-2 Detection with Pharyngeal Swab Specimen in COVID-19 Patients: A Retrospective Study. medRxiv.

[93] Wikramaratna P., Paton R.S., Ghafari M., Kingdom U. (2020). Estimating false-negative detection rate of SARS-CoV-2 by RT-PCR. medRxiv.

[94] Kucirka L.M., Lauer S.A., Laeyendecker O., Boon D., Lessler J. (2020). Variation in False Negative Rate of RT-PCR Based SARS-CoV-2 Tests by Time Since Exposure. medRxiv Prepr..

[95] Yedidag E.N., Koffron A.J., Mueller K.H., Kaplan B., Kaufman D.B., Fryer J.P., Stuart F.P., Asecassis M. (1996). Acyclovir triphosphate inhibits the diagnostic polymerase chain reaction for cytomegalovirus. Transplantation.

